# Behavioral aspects and neurobiological properties underlying medical cannabis treatment in *Shank3* mouse model of autism spectrum disorder

**DOI:** 10.1038/s41398-021-01612-3

**Published:** 2021-10-13

**Authors:** Shani Poleg, Emad Kourieh, Angela Ruban, Guy Shapira, Noam Shomron, Boaz Barak, Daniel Offen

**Affiliations:** 1 Sackler Faculty of Medicine, Human Molecular Genetics & Biochemistry, Felsenstein Medical Research Center, Tel-Aviv University, Tel Aviv, Israel; 2 The George S. Wise Faculty of Life Sciences, Tel Aviv University, Tel Aviv, Israel; 3 Sackler Faculty of Medicine, Tel Aviv University, Tel Aviv, Israel; 4 Sagol School of Neuroscience, Tel-Aviv University, Tel Aviv, Israel; 5 The School of Psychological Sciences, Faculty of Social Sciences, Tel Aviv University, Tel Aviv, Israel

**Keywords:** Clinical pharmacology, Autism spectrum disorders, Molecular neuroscience

## Abstract

Autism spectrum disorder (ASD) is a neurodevelopmental disease with a wide spectrum of manifestation. The core symptoms of ASD are persistent deficits in social communication, and restricted and repetitive patterns of behavior, interests, or activities. These are often accompanied by intellectual disabilities. At present, there is no designated effective treatment for the core symptoms and co-morbidities of ASD. Recently, interest is rising in medical cannabis as a treatment for ASD, with promising clinical data. However, there is a notable absence of basic pre-clinical research in this field. In this study, we investigate the behavioral and biochemical effects of long-term oral treatment with CBD-enriched medical cannabis oil in a human mutation-based *Shank3* mouse model of ASD. Our findings show that this treatment alleviates anxiety and decreases repetitive grooming behavior by over 70% in treated mutant mice compared to non-treated mutant mice. Furthermore, we were able to uncover the involvement of CB1 receptor (CB1R) signaling in the Avidekel oil mechanism, alongside a mitigation of cerebrospinal fluid (CSF) glutamate concentrations. Subsequently, RNA sequencing (RNA seq) of cerebellar brain samples revealed changes in mRNA expression of several neurotransmission-related genes post-treatment. Finally, our results question the relevancy of CBD enrichment of medical cannabis for treating the core symptoms of ASD, and emphasize the importance of the THC component for alleviating deficits in repetitive and social behaviors in ASD.

## Introduction

Autism spectrum disorder (ASD) is a neurodevelopmental disease with a wide spectrum of manifestation. The core symptoms of ASD are persistent deficits in social communication; restricted and repetitive patterns of behavior, interests, or activities. Although they are not defined as a core symptom, intellectual disabilities are often associated with ASD [1, 2]. Despite the high prevalence and the great social impact of ASD, there is still no cure for its core symptoms [3].

**Fig. 1:**
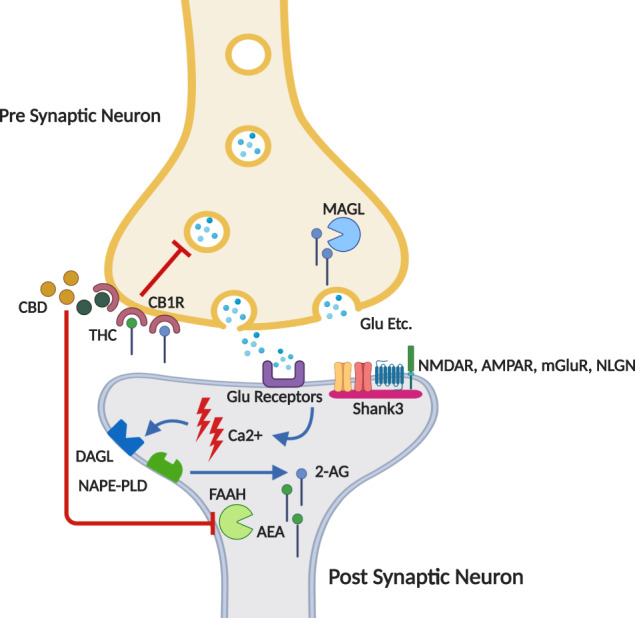
Schematic diagram of the EC system in glutamatergic synapse. **A** neurotransmitter (for example, glutamate) is released from the pre-synaptic neuron and binds its receptors in the post-synaptic neuron, causing post-synaptic depolarization and Ca2^+^ influx. The resultant increase in Ca2^+^ concentrations drives production of endocannabinoids such as anandamide (AEA) and 2-arachidonoylglycerol (2-AG) by Nacetylphosphatidylethanolamine-hydrolyzing phospholipase D (NAPE-PLD) and diacylglycerol lipase (DAGL) respectively, which in turn travel retrogradely from the post-synaptic neuron to the pre-synaptic neuron, where they bind CB1 receptors (CB1R). CB1R activation leads to suppression of further neurotransmitter release. AEA and 2-AG are degraded rapidly by FAAH and MAGL, respectively. CB1R can also be activated by exogenous agents such as the phytocannabinoids THC and CBD, which are found abundantly in the cannabis plant. Whilst THC has a strong affinity to the CB1R, CBD has a very low affinity to this receptor and its effects are mediated mostly by other means, such as inhibition of FAAH and elevating AEA levels. Also specified in this Figure is the SH3 and multiple ankyrin repeat domains 3 (SHANK3) protein, also known as proline-rich synapse-associated protein 2 (ProSAP2). SHANK3 is a scaffold protein that connects receptors to cytoskeletal signaling molecules and binds many ion channels, other scaffolding proteins, enzymes, and signaling molecules in the post-synaptic densities (PSDs). Since SHANK3 is concentrated in glutamatergic synapses, it interacts with all prominent glutmate receptors, such as NMDA, AMPA, and mGlu receptors. SHANK3 also indirectly interacts with Neuroligins (NLGN), a family of post-synaptic adhesion molecules. Most of these interactions are indirect and mediated by post-synaptic proteins such as GKAP, Homer PSD95 etc.

**Fig. 2:**
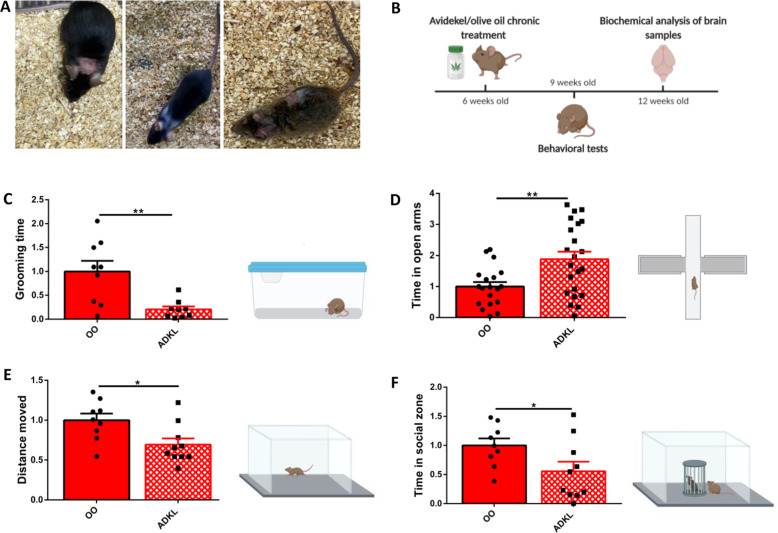
CBD-enriched medical cannabis treatment improves repetitive and anxiety behaviors, but not social deficits, in InsG3680 Shank3 mouse model of ASD. **A** InsG3680 *Shank3* mutant mice that were injured by excessive self-grooming repetitive behaviors. **B** Graphic description of the study design. Mice were treated twice a week with oral gavage of olive oil/Avidekel oil (control and experiment groups, respectively) for 3 consecutive weeks prior to the behavioral tests, and 1 h before each behavioral test. After termination of the behavioral tests, the mice were killed and brain samples went through biochemical analysis. **C** Repetitive grooming behavior test. Avidekel oil treatment reduced the amount of grooming performed by InsG3680 *Shank3* mutant mice (“ADKL”) in more than 70%, as compared with the InsG3680 *Shank3* mutant mice olive oil control (“OO”). Data are presented as mean ± SEM, and normalized to the olive oil control group. *N* = 18. **p < 0.01. Two-tailed *t*-test between groups. **D** Elevated plus maze test. Olive oil-treated InsG3680 *Shank3* mutant mice spent significantly less time exploring the open arms, as compared with Avidekel-oil-treated InsG3680 *Shank3* mutant mice. Data are presented as mean ± SEM, and normalized to the olive oil control group. *N* = 31 (two replications of the experiment). **p < 0.01. Two-tailed *t*-test between groups. **E** Open field test. Avidekel-oil-treated InsG3680 *Shank3* mutant mice walked a shorter distance in the arena, in comparison with the olive oil-treated InsG3680 *Shank3* mutant mice. Data are presented as mean ± SEM, and normalized to the olive oil control group. *N* = 19. *p < 0.05. Two-tailed t-test between groups. **F** Social approach test. Avidekel oil treatment decreased the time InsG3680 *Shank3* mutant mice spent in the social zone (namely, in close proximity to the stranger mouse cup) in the social approach test. Data are presented as mean ± SEM, and normalized to the olive oil control group. *N* = 19. *p < 0.05. Two-tailed *t*-test between groups.

Familial and twin studies indicate that ASD is, at least in part, a genetic neuropsychiatric disorder [4, 5]. Hence, there is a scientific interest in charactrizing reliable genetic mouse models of ASD, including the various *Shank3* mutant mouse models. SHANK3 protein is important for spinogenesis and synapse development and also plays a significant role in organizing the post-synaptic densities (PSDs) in glutamatergic synapses [6] (Fig. 1). Specifically in InsG3680 *Shank3* mutant mice, a point mutation that was identified in genetic screens of individuals with ASD causes a stop codon immediately after the G insertion in position 3680, thereby causing an almost complete loss of SHANK3 protein. This mouse model demonstrates prominent autistic-like behaviors, such as impaired social interaction, anxiety, and excessive repetitive self-grooming that leads to skin lesions in ~30% of the adult homozygous mice [7] (Fig. 2A).

The endocannabinoid (EC) system is a neuromodulating network that was previously shown to be implicated in ASD patients with co-morbidities such as anxiety and seizures [8, 9] (Fig. 1). The EC system regulates emotional responses, as well as behavioral reactivity to context and social interaction [8, 10]. In addition, the EC system plays an important role in the developing brain, as it maintains the inhibition/excitation ratio, regulates neural progenitor proliferation, and navigates axonal migration, among other functions [11–15].

Since EC imbalance is considered a possible mechanism leading to ASD’s core symptoms and morbidities [16], the option of treating individuals with ASD with medical cannabis is appealing. Cannabis plants contain over 100 different secondary compounds named phytocannabinoids, the two most abundant ones being Δ9-tetrahydrocannabinol (THC) and cannabidiol (CBD) [9, 17, 18]. CBD is considered a non-psychoactive phytocannabinoid and does not cause the euphoria associated with THC intake [9, 19–21]. However, evidence suggest that CBD has many other attributes, including anxiolytic [22, 23], anti-epileptic [24], and anti-inflammatory [19] effects. Importantly, enhanced neuroinflammatory state is also considered a possible pathophysiology that attributes to ASD. Specifically, the role of astrocytes and microglia in synaptic plasticity and neuroinflammation in ASD was previously described in the literature [25–33]. When treating individuals with ASD with medical cannabis, medical cannabis oils are the most prevalent route of administration. These oils are medications based on cannabis plant extracts that are administered orally. Furthermore, it is customary to designate CBD-enriched strains of cannabis that have low THC content for treating children and adolescents [24, 34, 35], given the general consensus that the psychoactive effects of high doses of THC are undesirable in these patients. Despite a large body of evidence supporting CBD-enriched medical cannabis for the treatment of individuals with ASD [34–37], large gaps remain in the understanding of the mechanisms of chronic treatment with medical cannabis in individuals with ASD.

Another neurological dysfunction that was linked previously to ASD is excessive activation of excitatory glutamatergic synapses combined with lack of glutamate uptake, which can cause excitability toxicity, also referred to as “excitotoxicity” [38–45]. Specifically, InsG3680 *Shank3* mutant mice show disruptions of glutamatergic signaling as compared to WT controls [46]. Since data suggest that excitotoxicity plays a role in the pathophysiology of ASD, reducing glutamate signaling might help treating ASD associated symptoms [47, 48].

Here, we investigated the behavioral and biochemical effects of CBD-enriched Avidekel oil (Tikun Olam Cannabis Pharmaceuticals, Tel Aviv, Israel) treatment on a InsG3680 *Shank3* mouse model of ASD (Fig. 2B). Our results indicate that Avidekel oil can alleviate excessive repetitive grooming and anxiety behaviors in InsG3680 *Shank3* mutant mice. Additionally, we demonstrate that these effects are mediated by CB1R activation, consequently causing a significant decrease in glutamate concentrations in the CSF, accompanied by differential expression of neurotransmission-related genes in the cerebellum as detected by RNA seq. Finally, we question the relevance of the CBD component of medical cannabis for treating autistic-like phenotypes in InsG3680 *Shank3* mouse model of ASD, and suggest that THC-based cannabis oil such as Erez oil (Tikun Olam Cannabis Pharmaceuticals, Tel Aviv, Israel) might be preferable, due to its favorable effect on social behavior in addition to alleviation of repetitive grooming behavior.

## Materials and methods

### Overview of the study

InsG3680 *Shank3* mutant mice in the experiment groups received a chronic treatment of 5 ml/kg Avidekel oil (CBD:THC ratio 20:1, 25 mg/kg CBD, 1 mg/kg THC). InsG3680 *Shank3* mutant mice in the control group received a chronic treatment of 5 ml/kg olive oil. A second cohort of naïve InsG3680 *Shank3* mutant mice was treated with either 5 ml/kg CBD oil (25 mg/kg CBD), Erez oil (1 mg/kg THC, no CBD), THC oil (1 mg/kg THC) or olive oil 5 ml/kg. The mice were treated by oral gavage twice a week for 3 consecutive weeks, one hour prior to each behavioral test or killing. In additional grooming tests, InsG3680 Shank3 mutant mice received an intraperitoneal (i.p) injection of 3 mg/kg of the CB1R antagonist AM-251, or 0.5 mg/kg of the CB1R agonist WIN55,212-2, 30 min before the behavioral test.

### Animals

Mice were treated as approved by the Tel Aviv University Institutional Animal Care and Use Committee (Ref No. 01-19-021). All mice were maintained in 12-h-light/12-h-dark conditions with ad libitum access to food and water. All mice were 6-weeks-old when the experiment started, and they were weighed once a week. Every effort was made to reduce the number of mice used and minimize their suffering. For detailed information regarding InsG3680 *Shank3* mouse model of ASD, please see supplementary information.

### Medical cannabis oils

CBD-enriched Avidekel oil that contains CBD:THC ratio of 20:1, THC-based Erez oil that contains THC and no CBD, and CBD oil that contains only CBD were kindly supplied by Tikun Olam Cannabis Pharmaceuticals (Tel Aviv, Israel). THC was kindly supplied by the professor Raphael Mechoulam lab (the Hebrew University of Jerusalem, Jerusalem, Israel). Mice in the control groups were treated with olive oil (Fluka, Sigma-Aldrich, 75343). Further information and HPLC Analysis of cannabis oils is available in the supplementary information (Table S1).

### Pharmacological compounds

The CB1R antagonist AM-251 (71670, Cayman Chemicals, Michigan, USA) and agonist WIN55,212-2 (10009023, Cayman Chemicals, Michigan, USA) were given intra-peritoneally.

### Behavioral tests

The mice underwent a series of behavioral tests, 1-h post oral gavage treatment. Detailed information regarding the behavioral tests is provided in the supplementary materials and methods, as well as raw data of the behavioral tests (Fig. S0).

### HPLC-FD analysis of glutamate and GABA in CSF

Glutamate and Gamma-Aminobutyric Acid (GABA) levels in the CSF were measured using high-performance liquid chromatography with fluorescence detection (HPLC-FD, 250 × 4.6 mm, 2.5 µl particle size column, Hypersyl Gold 5U column, Thermo Scientific, USA). Detailed information regarding CSF extraction and HPLC-FD protocols is shown in the supplementary information.

### Evaluation of cannabinoids in serum

Whole blood samples were collected after killing the mice by terminal bleeding. The samples were then incubated at room temperature for 30 min, following centrifugation at 1000 rcf for 10 min at 4 °C. The clear phase containing the serum was collected gently by pipetting and transferred to a new 1.5 ml tube for storage at −80 °C. Detailed information regarding experimental analysis of cannabinoids is shown in supplementary information.

### RNA sequencing of cerebellar brain samples

RNA from the right cerebellum of InsG3680 *Shank3* mutant mice was extracted using RNeasy Mini Kit (79254, Qiagen, Hilden, Germany). In short, mice designated for brain dissection were killed using CO2 1 h post oral gavage. Brains were removed quickly and dissected on ice for right cerebellum: the pons was separated from the brain using forceps. Immediately afterwards the cerebellum was dissected from the cortex using a scalpel. Then, the cerebellum was dissected in the midline and the right half was snap frozen in liquid nitrogen, then transferred to −80 °C until RNA extraction.

RNA extraction was performed following the RNeasy mini kit protocol. Afterwards, RNA sequencing was performed by Macrogen Europe NGS services. Raw sequencing data was trimmed and aligned to the GRCm38 assembly using STAR 2.7.3a [49]. Differential gene expression analysis was performed in R 3.6.3, using DESeq2 1.26 [50] and surrogate variable analysis with sva 3.34. Clustering of the samples was assessed using the principle component analysis (PCA) of variance stabilize transformed data [51]. A single outlier sample was omitted, resulting in decent clustering with notable batch effect (Fig. S1A). The batch effect was empirically corrected using surrogate variables produced using the sva package for R (v3.38), then incorporated to DESeq as covariates (Fig. S1B). The improved clustering is visualized using limma’s removeBatchEffect (Fig. S1C) (limma v3.46.0) [52].Raw data tables are supplied in supplementary materials (Tables S2–S3). Principal component analysis (PCA) is presented in supplementary (Fig. S1).

### Statistical analysis

The results are expressed as means± standard error mean (SEM). Statistical analysis was performed using unpaired Student’s *t* test for the direct comparison between two groups. Statistical analysis of data sets was carried out with the aid of GraphPad Prism 6.01 for Windows (Graphpad Software, CA, USA).

## Results

### CBD-enriched medical cannabis treatment improves repetitive and anxiety behaviors, but not social deficits, in InsG3680 Shank3 mouse model of ASD

In conducting this experiment, our prime goal was to assess the effects of long-term treatment with CBD-enriched medical cannabis on the autistic-like phenotypes of InsG3680 *Shank3* mouse model of ASD (Fig. 2B). In this experiment, mice were treated orally with either CBD-enriched Avidekel oil (25 mg/kg CBD, 1 mg/kg THC) or olive oil (5 ml/kg) twice a week, for 3 consecutive weeks, and 1 h prior to each behavioral test. The pivotal effect of the Avidekel oil treatment in InsG3680 *Shank3* mutant mice was in relieving excessive repetitive grooming behavior, as compared with the InsG3680 *Shank3* olive oil control group (Fig. 2C). Additionally, InsG3680 *Shank3* mutant mice treated with Avidekel oil spent significantly more time exploring the open arms in the elevated plus maze, indicating lower levels of anxiety (Fig. 2D). Furthermore, locomotor activity assessment in the open field test showed that InsG3680 *Shank3* mutant mice treated with Avidekel oil walked a significantly shorter distance than parallel mutant mice treated with olive oil, suggesting decreased locomotor activity post-treatment (Fig. 2E). Finally, in the social approach test, InsG3680 *Shank3* mutant mice treated with Avidekel oil spent less time sniffing the stranger mouse, in comparison with the olive oil treatment control group (Fig. 2F).

### CB1 receptors are involved in repetitive behavior alleviation as a result of Avidekel oil treatment in InsG3680 Shank3 mouse model of ASD

**Fig. 3:**
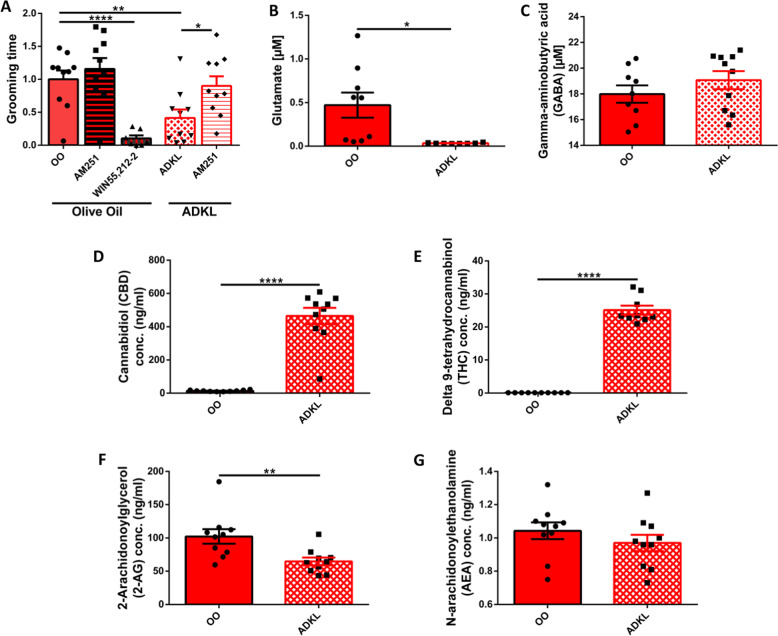
Revealing a possible mechanism for Avidekel oil in InsG3680 Shank3 mouse model of ASD. **A** CB1R mitigates the Avidekel-induced reduction in InsG3680 *Shank3* mutant mice repetitive grooming behavior. After treatment with CB1R antagonist AM-251 in addition to Avidekel oil, InsG3680 *Shank3* mutant mice have doubled the time they spent grooming, normalized to their baseline grooming level, which was determined after receiving solely Avidekel oil. Furthermore, CB1R blockade did not change the grooming behavior of the olive oil-treated InsG3680 *Shank3* mutant mice. In addition, treatment with the CB1R agonist Win 55,212-2 decreased significantly grooming behavior in olive oil-treated InsG3680 *Shank3* mutant mice. Data are presented as mean ± SEM. *N* = 20 (10 in each treatment group, Olive oil and Avidekel oil). *p < 0.05, **p < 0.01, ****p < 0.0001. Two-tailed paired *t*-test between groups. **B**, **C** HPLC-FD analysis of CSF neurotransmitters one hour post oral gavage. Avidekel oil treatment caused a significant decrease of over 90% in glutamate concentration in the CSF of InsG3680 *Shank3* mutant mice (**B**). No significant difference between the olive oil and Avidekel oil treatment groups was detected when regarding GABA concentrations (**C**). Data are presented as mean ± SEM. *N* = 20 (10 in each group). *p < 0.05. Two-tailed *t*-test between groups. **D**–**G** Liquid chromatography-mass spectrometry (LC-MS) measurements of serum cannabinoids one hour post oral gavage treatment in InsG3680 *Shank3* mutant mice. Measurement of exocannabinoids CBD (**D**) and THC (**E**) concentrations in serum indicated high concentrations of CBD and THC in the Avidekel oil treatment group. Furthermore, the results show that CBD concentration in the Avidekel oil treatment group is approximately 20 times higher than THC, in concordance with the CBD:THC ratio that was reported by the manufacturer. Measurement of serum endocannabinoids 2-AG (**F**) and anandamide/AEA (**G**) revealed a significant decrease of approximately 40% in 2-AG concentration, while there was no significant decrease in the concentration of anandamide in the serum. Data are presented as mean ± SEM. *N* = 20 (10 in each group). **p < 0.01, ****p < 0.0001. Two-tailed *t*-test between groups.

Given the prominent role of CB1R in the brain’s EC system, we wanted to ascertain whether CB1 receptor signaling is involved in the mechanism of Avidekel oil alleviation of InsG3680 *Shank3* mutant mice’s repetitive grooming behavior. Blockade of CB1 receptors using AM-251 (3 mg/kg, i.p., 30 min prior to the grooming test) in InsG3680 *Shank3* mutant mice treated with Avidekel oil significantly increased their grooming behavior, in comparison with their baseline level, which was determined after merely a single treatment of Avidekel oil (Fig. 3A). This effect was not significant in the olive oil treatment group. Furthermore, enhancement of CB1R signaling using the CB1R agonist WIN55,212-2 (0.5 mg/kg, i.p., 30 min prior to the grooming test) decreased significantly the repetitive grooming behavior performed by InsG3680 *Shank3* mutant mice treated with olive oil (Fig. 3A).

### CBD-enriched medical cannabis treatment decreased glutamate concentration in the CSF of InsG3680 Shank3 mutant mice

In order to elucidate a molecular explanation for the behavioral changes observed in InsG3680 *Shank3* mutant mice as a result of Avidekel oil treatment, and since CB1R activation results in decreased release of glutamate in glutamatergic synapses, we conducted a HPLC-FD analysis of InsG3680 *Shank3* mutant mice CSF for glutamate and GABA. Our results revealed that treatment with Avidekel oil significantly decreased glutamate concentration in the CSF one hour post-treatment, showing a decrease of over 90% compared with the olive oil control group (Fig. 3B). Nevertheless, no significant change in GABA levels was detected in the CSF (Fig. 3C).

### CBD-enriched medical cannabis treatment influences concentrations of cannabinoids in the serum of InsG3680 Shank3 mutant mice

In a series of liquid chromatography-mass spectrometry (LC-MS) tests, concentrations of exocannabinoids as well as endocannabinoids in the serum of InsG3680 *Shank3* mutant mice were measured, so as to evaluate the effect of Avidekel oil treatment on the endocannabinoid system. Treatment with Avidekel oil led to high concentrations of CBD and THC in the serum one hour post-treatment, and the CBD:THC ratio of 20:1 that was reported by the manufacturer was preserved (Fig. 3D, E). Also, we identified that the endocannabinoid 2-AG concentration in the serum of InsG3680 *Shank3* mutant mice treated with Avidekel oil decreased by almost 40% (Fig. 3F). However, no significant decrease in anandamide concentration was detected (Fig. 3G).

### CBD-enriched cannabis oil modifies mRNA expression in the cerebellum of InsG3680 Shank3 mutant mice

**Fig. 4:**
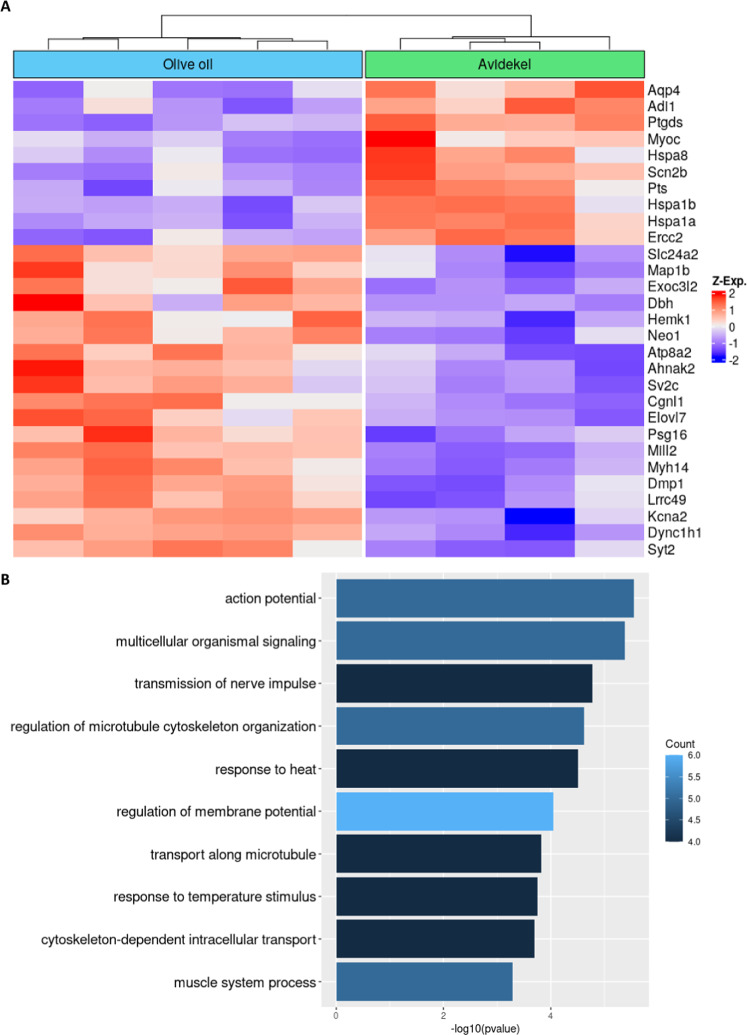
Avidekel oil treatment caused significant changes in mRNA expression in the InsG3680 Shank3 mutant mice cerebellum. **A** Heatmap of gene expression (batch-corrected, *Z*-score normalized gene counts) of the top 30 significantly differentially expressed protein coding genes (FDR < 0.05). **B** Significant gene ontology terms enriched in the differentially expressed genes, sorted by log-transformed *p*-value and colored according to the number of enriched genes for each term.

To study the transcriptomic alterations as a result of Avidekel oil treatment in comparison with olive oil treatment in InsG3680 *Shank3* mutant mice, we performed RNA seq of cerebellar brain samples. First of all, since cerebellar abnormalities were previously linked to excessive repetitive behaviors [53–55], and the most prominent effect of Avidekel oil treatment in InsG3680 *Shank3* mutant mice was the alleviation of excessive repetitive grooming behavior (Fig. 2C). In addition, our previous studies strengthen the claim that cerebellar brain regions are implicated in different mouse models of ASD [56–58]. Our results (as described profoundly in Table 1 and Fig. 4), revealed significant differential expression of several autism-related genes, such as aquaporine-4 (Aqp4)), dynein cytoplasmic 1 heavy chain 1 (Dync1h1), neogenin1 (Neo1), and dopamine beta-hydroxylase (Dbh). In addition, genes coding for ion channels such as sodium voltage-gated channel beta subunit 2 (Scn2b), sodium voltage-gated channel alpha subunit 8 (Scn8a), and potassium voltage-gated channel subfamily a member 2 (Kcna2) were also differentially expressed in the cerebellum of InsG3680 *Shank3* mutant mice treated with Avidekel oil compared with controls treated with olive oil. Furthermore, changes in mRNA expression of heat shock proteins such as heat shock protein family a member 1a (Hspa1a) and heat shock protein family a member 1b (Hspa1b) were also observed in the Avidekel oil group compared with the olive oil control group (Fig. 4A). However, gene ontology enrichment analysis revealed that the most significant change was in gene ontologies related to regulation of membrane potential, action potential and transmission of nerve impulse (Fig. 4B). Overall, our RNA seq results support the hypothesis that the cannabis-induced alleviation of repetitive grooming behavior in InsG3680 *Shank3* mutant mice is mediated by neurotransmission regulation.

**Table 1:** Table showing top 30 most significantly differentially expressed protein coding genes.

Symbol	Description	FoldChange	*p*-value	FDR
Ptgds	Prostaglandin D2 synthase (brain)	0.77	0	1.2E-10
Hspa1b	Heat shock protein 1B	0.47	2.7E-09	1.4E-05
Hspa1a	Heat shock protein 1A	0.55	5.2E-09	2.1E-05
Sv2c	Synaptic vesicle glycoprotein 2c	1.4	4.1E-08	0.00013
Adi1	Acireductone dioxygenase 1	0.74	5.8E-08	0.00015
Lrrc49	Leucine rich repeat containing 49	1.24	2.8E-07	0.00063
Hspa8	Heat shock protein 8	0.8	1.2E-06	0.0024
Exoc3l2	Exocyst complex component 3-like 2	6.05	1.6E-06	0.0028
Ercc2	Excision repair cross-complementing rodent repair deficiency, complementation group 2	0.67	2E-06	0.0029
Myh14	Myosin, heavy polypeptide 14	1.22	2E-06	0.0029
Scn2b	Sodium channel, voltage-gated, type II, beta	0.84	2.4E-06	0.0032
Map1b	Microtubule-associated protein 1B	1.24	3.6E-06	0.004
Mill2	MHC I like leukocyte 2	1.93	4.2E-06	0.0044
Aqp4	Aquaporin 4	0.82	4.6E-06	0.0045
Hemk1	HemK methyltransferase family member 1	1.6	1.2E-05	0.011
Pts	6-pyruvoyl-tetrahydropterin synthase	0.7	1.6E-05	0.014
Elovl7	ELOVL family member 7, elongation of long chain fatty acids (yeast)	1.34	1.7E-05	0.014
Dbh	Dopamine beta hydroxylase	3.54	2E-05	0.016
Syt2	Synaptotagmin II	1.15	2.3E-05	0.017
Dync1h1	Dynein cytoplasmic 1 heavy chain 1	1.13	3.5E-05	0.025
Neo1	Neogenin	1.19	3.9E-05	0.027
Atp8a2	ATPase, aminophospholipid transporter-like, class I, type 8 A, member 2	1.23	5.7E-05	0.036
Kcna2	Potassium voltage-gated channel, shaker-related subfamily, member 2	1.14	6.8E-05	0.042
Ahnak2	AHNAK nucleoprotein 2	1.78	7.6E-05	0.045
Myoc	Myocilin	0.43	9.2E-05	0.047
Slc24a2	Solute carrier family 24 (sodium/potassium/calcium exchanger), member 2	1.16	8.6E-05	0.047
Dmp1	Dentin matrix protein 1	1.39	9E-05	0.047
Psg16	Pregnancy specific glycoprotein 16	1.47	8.5E-05	0.047
Cgnl1	Cingulin-like 1	1.22	0.0001	0.05
Scn8a	Sodium channel, voltage-gated, type VIII, alpha	1.13	0.00012	0.055
Plcl1	Phospholipase C-like 1	1.25	0.00014	0.06
Glra1	Glycine receptor, alpha 1 subunit	1.58	0.00014	0.06

### CBD alone is not responsible for the behavioral effects of Avidekel oil on InsG3680 Shank3 mutant mice’s behavior

**Fig. 5:**
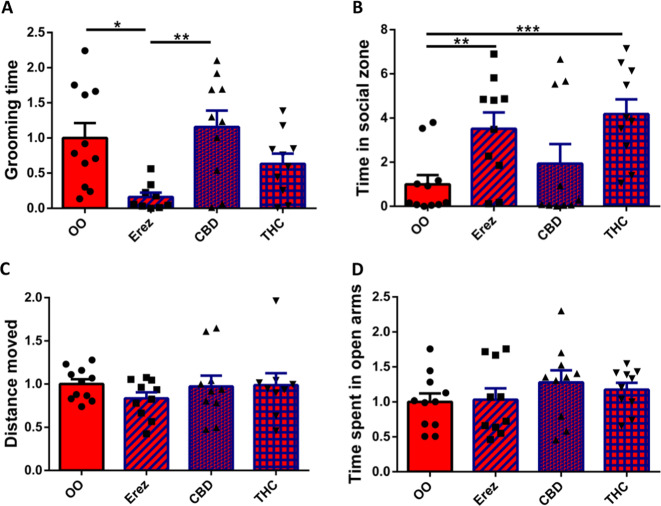
CBD alone is not responsible for the behavioral effects of Avidekel oil on InsG3680 Shank3 mutant mice behavior. **A** Repetitive grooming behavior test. Erez oil-treated InsG3680 *Shank3* mutant mice spent one-fifth of the time grooming, compared to the olive oil-treated InsG3680 *Shank3* control group. Furthermore, while CBD treatment did not affect InsG3680 *Shank3* mutant mice repetitive grooming behavior, THC treatment caused an insignificant trend of decrease in this behavior. **B** Social approach test. Both Erez oil and THC-treated InsG3680 *Shank3* mutant mice spent significantly more time in the social zone in the social approach test, normalized to the olive oil-treated InsG3680 *Shank3* mutant mice. Again, no significant change was observed in the CBD treatment group. **C** Open field test. InsG3680 *Shank3* mutant mice showed no change in the locomotor activity as measured in the open field test after treatment with either CBD, THC, or Erez oil, normalized to the olive oil control group. **D** Elevated plus maze test. InsG3680 *Shank3* mutant mice treated with either CBD, THC, or Erez oil did not manifest changes in the time spent exploring the open arms in the elevated plus maze test, normalized to the olive oil-treated InsG3680 *Shank3* mutant mice. Results are normalized to the InsG3680 *Shank3* olive oil control. All data are presented as mean ± SEM. *N* = 40 (10 in each group). *p < 0.05, **p < 0.01, ***p < 0.0006. Two-tailed *t*-test between groups.

To clarify whether CBD enrichment of medical cannabis is necessary for the alleviation of the autistic-like symptoms of InsG2680 *Shank3* mutant mice, we evaluated the effect of THC-based Erez oil on mice behavior compared with the behavior of controls treated with olive oil. We treated InsG3680 *Shank3* mutant mice orally twice a week for 3 consecutive weeks, as well as one hour prior to each behavioral test, with either olive oil or Erez oil (1 mg/kg THC, no CBD). Our results show that compared to olive oil treatment, Erez oil treatment decreased significantly the repetitive grooming behavior performed by InsG3680 *Shank3* mutant mice (Fig. 5A) and also improved significantly their social behavior in the social approach test (Fig. 5B). Nevertheless, Erez oil treatment did not change the InsG3680 *Shank3* mutant mice behavior in the open field (Fig. 5C) and elevated plus maze tests (Fig. 5D). These results led us to test the influence of isolated CBD (25 mg/kg, as found in Avidekel oil) and THC (1 mg/kg, as found in both Avidekel and Erez oils) oils on InsG3680 *Shank3* mutant mice behavior. Again, InsG3680 *Shank3* mutant mice were treated orally twice a week for 3 consecutive weeks, and afterwards one hour prior to each behavioral test, with either THC or CBD. We found that CBD oil alone does not change significantly the anxiety, social, locomotor and repetitive grooming behaviors performed by InsG3680 *Shank3* mutant mice compared to olive oil treatment (Fig. 5A–D). However, treatment with pure THC (1 mg/kg) demonstrated an insignificant trend of decrease in repetitive grooming behavior (Fig. 5A), alongside a significant improvement in the mice’s social behavior (Fig. 5B). As such, these results suggest that CBD enrichment of medical cannabis is not necessary for the alleviation of autistic-like phenotypes in InsG3680 *Shank3* mutant mice.

### THC-based medical cannabis alleviation of autistic-like phenotypes in InsG3680 Shank3 mouse model of ASD is also mediated by CB1R

Since THC-based Erez oil decreased repetitive grooming behavior in InsG3680 *Shank3* mutant mice similarly to Avidekel oil, we sought to test whether these two medical cannabis oils have a shared mechanism. Our results showed that CB1R blockade by the CB1R antagonist AM251 (3 mg/kg, i.p.) increased the amount of grooming performed by InsG3680 *Shank3* mice treated with Erez oil, as compared to their baseline level, which was determined after merely one single treatment with Erez oil (1 mg/kg THC) (Fig. S2). Moreover, treatment with Erez oil decreased glutamate and increased GABA concentrations in the CSF of InsG3680 *Shank3* mutant mice significantly, compared to olive oil treatment (Fig. S3A, B). Additionally, LC-MS analysis of serum samples indicated that treatment with Erez oil resulted in a detectable concentration of THC, but not CBD, in the serum one hour post-treatment (Fig. S3C, D). Furthermore, serum analysis revealed significant decreased concentrations of both 2-AG and anandamide (Figures S3E, F).

### Chronic treatment with medical cannabis oils did not impair the well-being of InsG3680 Shank3 mutant mice

In order to assess the effects of long-term treatment with medical cannabis oils on well-being in the InsG3680 *Shank3* mouse model of ASD, we tested the mice in the Y maze and forced swimming tests after 6 weeks of semiweekly treatment with olive, Avidekel, or Erez oils. In addition, the mice received treatment one hour prior to the behavioral test. Our results show that medical cannabis treatments did not harm the cognitive function of InsG3680 *Shank3* mutant mice compared to the olive oil control group as detected in the Y maze test (Fig. S4A). Furthermore, medical cannabis treatment did not increase susceptibility to negative mood of InsG3680 *Shank3* mice as tested in the forced swim test, compared with the control mice treated with olive oil (Fig. S4B). Finally, there was no significant difference in the weight of InsG3680 *Shank3* mice that were treated with medical cannabis oils, in comparison with mice treated with olive oil, suggesting that the treatment did not interfere with the mice’s appetite and physical maturation (Fig. S5).

### The beneficial effects of medical cannabis treatment on the repetitive grooming behavior of InsG3680 Shank3 mutant mice are acute

Throughout this study, InsG3680 *Shank3* mutant mice were treated with medical cannabis or olive oils both chronically and prior to the behavioral tests. In order to understand the contribution of chronic treatment with medical cannabis oils to the alleviation of the autistic-like behaviors of InsG3680 *Shank3* mutant mice, a naïve cohort of mice were treated with the appropriate oils exclusively one hour prior to a grooming behavioral test (Fig. S6). Our results indicated that a single treatment with medical cannabis oils (Avidekel and Erez, same doses as described) significantly decreased the repetitive grooming behavior performed by InsG3680 *Shank3* mutant mice, compared with the olive treated mice. Considering the dramatic effect of the acute treatment with medical cannabis oils on the measured autistic-like behavior, we conclude that the alleviation of the autistic-like phenotypes of InsG3680 *Shank3* mutant mice is mainly due to acute effects of the treatment, while the chronic treatment has little influence on the behavior.

## Discussion

Recently, interest is growing in CBD-enriched cannabis-based treatments for ASD. However, treating individuals with ASD with medical cannabis raises medical and ethical questions, since a large portion of the patients are young children [59]. During young childhood and adolescence, multiple neurological systems, such as the glutamatergic, dopaminergic and endocannabinoid systems, rearrange and maturate [60]. Data show that some cannabinoids, THC among them, might harm the developing brain [60–62]. Hence, efforts are being made to find a well-balanced medical cannabis-based treatment for individuals with ASD, which on one hand would alleviate their symptoms, and on the other hand would not cause them any further neurological harm.

Upon commencing this study, our main goal was to assess the behavioral and biochemical effects of chronic treatment with CBD-enriched Avidekel oil on the InsG3680 *Shank3* mouse model of ASD. As previously described, disruptive behaviors such as tantrums, aggression and self-injury are prevalent among children and adolescents with ASD [9, 63]. It was also reported that CBD-enriched cannabis oil was beneficial in treating some of these maladaptive behaviors in children with ASD [37]. Our results demonstrated a CB1R signaling involvement in the Avidekel oil alleviation of InsG3680 *Shank3* mutant mice’s excessive repetitive grooming behavior. This finding led us to study the downstream effects on the excitation/inhibition balance, which were evaluated by measuring CSF concentrations of GABA and glutamate. Importantly, this test is not a direct measurement of brain E/I balance, however it serves as a gross peripheral indication for it, with clinical applications.

As previously described, a delicate balance between excitatory glutamate and inhibitory GABA neurotransmitters is crucial for proper development and functioning of the brain, since it maintains excitability, integrity and synaptic plasticity [47]. According to the “Intense World Theory” of ASD, enhanced excitation/inhibition (E/I) ratio offers a neuropathologic explanation for ASD and underlies numerous autistic behavioral phenotypes [2, 38–45, 64–68]. In addition, several hyperexcitable brain regions implicated in this theory express CB1R abundantly, marking CB1R as a therapeutic target for this hyperexcitation [69–71], since the main result of CB1R activation is the reduction of pre-synaptic glutamate [38, 72]. Using HPLC-FD, we revealed a significant decrease in glutamate concentration in the CSF of InsG3680 *Shank3* mutant mice treated with Avidekel oil compared with the control mice treated with olive oil. This result indicates a decrease in extra-cellular glutamate concentration, implying a general decrease in brain excitation, which in turn led to decreased production and release of the endocannabinoid 2-AG, as detected in serum samples using LC-MS. Since endocannabinoids are produced per demand in the synapse [73], in case of a significant decrease in glutamate levels the demand for endocannabinoids also decreases and lower concentrations of circulating endocannabinoids are found in the serum.

In addition to mitigation of excitatory signaling as detected in the CSF and serum, our RNA seq results also demonstrated that Avidekel oil treatment modifies mRNA expression of neurotransmission-related genes in cerebellar brain samples of InsG3680 *Shank3* mutant mice. As mentioned, most of the differentially expressed genes were part of gene ontologies related to neuronal transmission, such as regulation of membrane potential, action potential, and transmission of nerve impulse. Since morphologic, compositional and pathological cerebellar changes were previously linked to excessive repetitive behaviors in ASD [53–57], altered expression of neurotransmission-related genes in this brain area might contribute to alleviation of excessive repetitive grooming behavior in InsG3680 *Shank3* mutant mice.

Interestingly, we also identified differential mRNA expression of several autism-related genes (*Dbh*, *Aqp4*, *Neo1*, and *Dync1h1* [74–80]), which might contribute to alterations in the mice autistic-like phenotypes. Finally, our RNA seq results detected decreased expression of heat shock proteins genes such as *Hspa1a* and *Hspa1b*. As chaperone molecules, these heat shock proteins play a role in maintaining the homeostasis of the cell by assisting in folding and degradation of peptides and proteins [81, 82]. During periods of cell stress and in some pathologies, the expression of these proteins increases, to assist the cell while coping with larger amounts of misfolded proteins and aggregations. Significantly lower mRNA expression of these heat shock proteins, as observed in the Avidekel-oil-treated group, might be a result of decreased cell stress after treatment and may represent additional positive effect of medical cannabis treatment in InsG3680 *Shank3* mouse model of ASD.

As mentioned earlier, physicians and researchers tend to prefer CBD-enriched medical cannabis products for the treatment of young patients with ASD. However, our results indicate that the CBD enrichment of medical cannabis is not necessary for treating the autistic-like phenotypes of InsG3680 *Shank3* mutant mice. Furthermore, our results suggest that THC-based medical cannabis oil is preferable for that purpose, since it improved InsG3680 *Shank3* mutant mice’s social behavior in addition to decreasing their excessive grooming behavior. However, Erez oil treatment did not cause anxiolytic effect or hypolocomotion in InsG3680 *Shank3* mutant mice, unlike Avidekel oil treatment; it is possible that large amounts of CBD combined with THC, as found in Avidekel oil, are required for inducing hypolocomotion and anxiolytic effects, both of which are well known effects of treatment with cannabinoids [22, 83–87]. In addition, based on our results, medical cannabis compounds such as Avidekel and Erez have a greater impact on InsG3680 *Shank3* mutant mice’s autistic-like phenotypes than purified CBD and THC, probably due to an “entourage effect” caused by other components found aplenty in medical cannabis extracts [88–90]. Notably, other studies showed that treatment with THC in extremely low doses did have a long lasting significant effect on mice’s behavior, however it was mainly relevant to cognitive functions rather than autistic-like behaviors [91–93].

Since THC-based Erez oil showed impact similar to CBD-enriched Avidekel oil on the InsG3680 *Shank3* mutant mice’s repetitive grooming behavior, we hypothesized that these two medical cannabis oils have a shared mechanism. Indeed, CB1R blockade did interfere with the Erez oil-induced reduction in repetitive grooming behavior performed by InsG3680 *Shank3* mutant mice. As expected, treatment with Erez oil also decreased the excitatory tone in the brains of InsG3680 *Shank3* mutant mice, compared with olive oil treatment, leading to significantly decreased glutamate and increased GABA concentrations in the CSF as detected by HPLC-FD analysis. However, the decrease in glutamate concentration after Erez oil treatment was less robust compared to Avidekel oil (50 and 90%, respectively). This might serve as an explanation for the decreased locomotion activity observed only after Avidekel oil treatment, which could harm the Avidekel oil-treated mice’s performance in the social behavior test. In addition, a significant decrease in 2-AG and anandamide concentrations in the serum of InsG3680 mice treated with Erez oil compared to those treated with olive oil also suggests a robust activation of CB1R 1 h post-treatment.

With respect to the fact that individuals with ASD are often children and young adults, it is vital to ascertain that chronic medical cannabis treatment does not interfere with physical development as well as mental and cognitive functions. Subsequently, our results show that long-term medical cannabis treatment did not affect the weight of InsG3680 *Shank3* mutant mice. In addition, our findings in the Y maze and forced swim tests indicated that long-term treatment with medical cannabis oils did not harm significantly the working memory and motivation of InsG3680 *Shank3* mutant mice compared to those of control mice treated with olive oil.

Finally, we were able to ascertain that the beneficial effect of medical cannabis treatment on the excessive grooming behavior of InsG3680 *Shank3* mutant mice is acute, for the most part. When added to our previously described results, this information further fortifies our hypothesis that THC activation of CB1R signaling has a major role in mediating the behavioral effects of the treatment in these mice. Moreover, previous animal studies showed that an acute treatment with THC and synthetic CB1R agonizts can decrease repetitive grooming behavior [94–96].

Our research presents a profound investigation of the influences of chronic treatment with medical cannabis oil in the InsG3680 *Shank3* mutant mouse model of autism. However, we note that this study has several limitations. First, InsG3680 *Shank3* is a genetic model for ASD, whilst among human individuals with ASD there are numerous presumed causes for ASD. In addition, despite the fact that ASD is far more common in males, females are also affected. Therefore, it is possible that our findings do not necessarily apply to all ASD mouse models, let alone human patients.

In summary, our study presents an extensive investigation of the behavioral and biochemical influences of medical cannabis treatment on a well-characterized genetic mouse model of ASD. We were able to demonstrate a significant decrease in the repetitive grooming and anxiety behaviors after treatment with CBD-enriched Avidekel oil and to unearth the involvement of CB1R and glutamate in this process. Furthermore, RNA seq results also indicated changes in the expression of neurotransmission and autism-related genes. Nevertheless, our results question the necessity of high doses of CBD in medical cannabis oils designated for alleviating ASD core symptoms, and suggest that a medical cannabis oil that contains relatively small doses of THC is preferable, due to an additional plausible effect on social behavior. Although much remains unclear regarding the short- and long-term effects of chronic treatment with medical cannabis in individuals with ASD, this study contributes to the accumulating scientific knowledge on this topic and should help physicians in tailoring a suitable cannabis-based treatment for their ASD patients, taking into account the patients’ phenotype as well as the medical cannabis characteristics.

## Supplementary information

Supplementary materials contents explanation

Supplementary materials and methods

Suppelmentary table 1- cannabis oils HPLC reports

Supplementary table 2- RNA seq genes

Supplementary table 3- RNA seq GO

Figure S0 - behavioral tests raw data

Figure S1- PCA RNA-Seq

Figure S2 - Erez CB1R blockade

Figure S3 - Erez Glu GABA CSF and serum cannabinoids

Figure S4 - well being after treatment with medical cannabis

Figure S5 - weight

Figure S6 - acute treatment only

Supplementary legends
