# Supplementary material for: Behavioral aspects and neurobiological properties underlying medical cannabis treatment in Shank3 mouse model of autism spectrum disorder
Source: Transl Psychiatry. 2021 Oct 13;11:524. doi: 10.1038/s41398-021-01612-3 (PMC8514476; doi:10.1038/s41398-021-01612-3)
Supplement: Supplementary file 1 — Supplementary materials contents explanation [file 41398_2021_1612_MOESM1_ESM.docx]

**Supplementary Materials**

| **File type** | **Content** | **File title** |
| --- | --- | --- |
| Word | Complete description of all the materials and methods used in the study | Supplementary materials and methods |
| Word | HPLC reports of all commercial medical cannabis oils that were supplied by Tikun Olam pharmaceuticals. | Supplementary table 1-cannabis oils HPLC reports |
| Excel | Raw RNA-seq data (differentially expressed genes) | Supplementary table 2 - RNA seq genes |
| Excel | Raw RNA-seq data (GO- gene ontologies) | Supplementary table 3 - RNA seq GO |
| Power point | figure | Supplementary figure 0 -  Raw data graphs |
| Power point | figure | Supplementary figure 1 -  PCA of RNA-seq |
| Power point | figure | Supplementary figure 2 -  Erez CB1R blockade |
| Power point | figure | Supplementary figure 3 - Erez glutamate GABA CSF and serum cannabinoids |
| Power point | figure | Supplementary figure 4 -well-being after treatment with medical cannabis |
| Power point | figure | Supplementary figure 5 - weight |
| Power point | figure | Supplementary figure 6 - acute treatment only |
| Word | Figure legends | Supplementary legends |
