# Supplementary material for: Behavioral aspects and neurobiological properties underlying medical cannabis treatment in Shank3 mouse model of autism spectrum disorder
Source: Transl Psychiatry. 2021 Oct 13;11:524. doi: 10.1038/s41398-021-01612-3 (PMC8514476; doi:10.1038/s41398-021-01612-3)
Supplement: Supplementary file 2 — Supplementary materials and methods [file 41398_2021_1612_MOESM2_ESM.docx]

**Supplementary materials and methods for**

**Behavioral Aspects and Neurobiological Properties Underlying Medical Cannabis Treatment in Shank3 Mouse Model of Autism Spectrum Disorder**

1. **Materials and Methods**
   1. Overview of the study

InsG3680 *Shank3* mutant mice in the experiment groups received a chronic treatment of 5 ml/kg Avidekel oil (CBD:THC ratio 20:1, 25 mg/kg CBD, 1 mg/kg THC). InsG3680 *Shank3* mutant mice in the control groups received a chronic treatment of 5 ml/kg Olive Oil. A second cohort of naïve InsG3680 *Shank3* mutant mice was treated with either 5 ml/kg CBD oil (25 mg/kg CBD), Erez oil (1 mg/kg THC, no CBD), THC oil (1 mg/kg THC) or olive oil 5 ml/kg. The mice were treated by oral gavage twice a week for three consecutive weeks, and one hour prior to each behavioral test or sacrifice. In additional grooming tests, InsG3680 Shank3 mutant mice received an intraperitoneal (i.p) injection of 3 mg/kg of the CB1R antagonist AM-251, or 0.5 mg/kg of the CB1R agonist WIN55,212-2, 30 minutes before the behavioral test.

- 1. Animals

Mice were treated as approved by the Tel Aviv University Institutional Animal Care and Use Committee (Ref No. 01-19-021). All mice were maintained in 12-h-light/12-h-dark conditions with ad libitum access to food and water. All mice were six weeks old when the experiment has started. The animals were weighed once a week for the entire experimental period. Every effort was made to reduce the number of mice used and minimize their suffering. Animals were allocated to groups randomly. For the behavioral test, groups contained n≥9 due to high variance. the analysis of the results was not blinded.

- - 1. InsG3680 Shank3 Mouse Model of ASD

InsG3680 Shank3 Heterozygous breeding pairs were a generous gift from Gouping Feng, PhD (McGovern Institute of Brain Research, Department of Brain and Cognitive Sciences, MIT, USA) and were bred at the FMRC facility (Beilinson Hospital, Petah-Tikvah, Israel). Heterozygous InsG3680 Shank3 females were bred with Homozygous InsG3680 Shank3 males. The offspring genotype was assessed using tissue samples obtained via tail piece (AccuStart II Mouse Genotyping Kit - Quanta Bio, Beverly, MA, USA). PCR was performed following the AccuStart II protocol, with suitable forward and reverse primers (Forward 5’- tgcaaacccgagactctgagagagg-3’, Reverse 5’-agcgaataccagctctggctcctcc-3’). Only male Homozygous offspring were used in the experiment.

- 1. Medical Cannabis oils

CBD-enriched Avidekel oil that contains CBD:THC ratio of 20:1, THC-based Erez oil that contains THC and no CBD and CBD oil that contains only CBD were kindly supplied by Tikun Olam Cannabis Pharmaceuticals (Tel Aviv, Israel). THC was kindly supplied by professor Raphael Mechoulam lab (The Hebrew University of Jerusalem, Jerusalem, Israel). Mice in the control group were treated with olive oil (Fluka, Sigma-Aldrich, 75343). HPLC Analysis of cannabis oils available in supplementary information (Table S1).

- 1. Pharmacological compounds

The CB1R antagonist AM-251 (71670, Cayman chemicals, Michigan, USA) and agonist WIN55,212-2 (10009023, Cayman chemicals, Michigan, USA) were given intra-peritoneally.

- 1. Behavioral tests
     1. Repetitive behavior - grooming

This test was performed to assess the repetitive grooming behavior. The mouse was placed in a 40X20X20 cm empty home cage and videotaped for 20 minutes. The first 10 minutes served as an acclimation of the mouse to the new environment and were not part of the analysis. The duration of grooming behavior in the last 10 minutes of the test was measured and quantified using Cowlog video coding software, and post analyses was done using Python software. Python code was written by a third party and it is not available online.

- - 1. Elevated plus maze

Elevated plus maze test was performed to assess anxiety related behavior. The test was performed in a plus-shaped maze containing two dark and enclosed arms (20lx) and two lit and open arms (60lx), elevated 100 cm above ground. The arms are 30×5 cm with a 5×5 cm center area, and the walls of the closed arms are 40 cm high. The mouse was placed in the center of the maze facing a closed arm, and videotaped for 5 min. The time spent in the open and closed arms of the maze was scored using Ethovision video tracking system (v11.5, Noldus, Wageningen, Netherlands). Percent of the time spent in the open arms was calculated (open/(open+closed) x100).

- - 1. Open field

Open field test was performed to assess locomotor activity. Each mouse was individually placed in an unfamiliar cubical shaped arena (50×50x50 cm) facing a wall, and was videotaped for 10 minutes. The Total distance traveled by the mouse was measured and scored using the Ethovision video tracking system (v11.5, Noldus, Wageningen, Netherlands).

- - 1. Social approach

Social approach test was performed to assess social behavior. The mouse was placed individually in an open field arena as described above, for acclimation of total 10 minutes. Immediately afterwards a matched age and weight male Stranger mouse was placed in the center of the arena inside a wired cup, and the mice were videotaped for 10 minutes. The stranger mice were habituated by placing them inside a wire cup for two sessions of 30 minutes each, one day before the test. The analysis for time spent in close proximity to the stranger mouse was carried out using the Ethovision video tracking system (v11.5, Noldus, Wageningen, Netherlands). Time spent in close proximity to the stranger mouse was defined as the amount of time in which the tested mouse spent in the "social zone", a circular area surrounding the wired cup containing the stranger mouse. The size of the social zone area was defined as twice the size of the wired cup.

- - 1. Y maze

Forced alteration Y maze was performed to assess spatial memory. The test was conducted in a white Perspex Y-shape maze apparatus with 38x5x15 cm arms. The mouse was placed in the entrance arm of the Y maze, while one arm of the maze is closed (this arm is designated as the novel arm). After a training session of five minutes the mouse was removed from the maze, and the maze was thoroughly cleaned. Five minutes after the termination of the training session, the mouse was placed again in the entrance arm of the maze, with the novel arm open for entry. The mouse was recorded and the video was analyzed using the Ethovision video tracking system (v11.5, Noldus, Wageningen, Netherlands). The percent of the time spent in the novel arm was calculated as (amount of time spent in the novel arm /the amount of time spent in all arms) x100.

- - 1. Forced swimming

Forced swimming test was performed to assess susceptibility to negative mood. The mouse was placed in a 30 cm diameter plastic cylinder filled with water (water temp was ~25°C) and was taped for six minutes. Manual analysis of the time that the mouse spent swimming was performed using Cowlog video coding software, and post analyses was done using Python software.

- 1. HPLC-FD Analysis of glutamate and GABA in CSF

InsG3680 Shank3 mutant mice CSF was aspirated (Vivox, Nesher, Israel). In brief, mice were anesthetized using Ketamine-Xylazine anesthesia solution (ketamine 100 mg/kg, xylazine 10 mg/kg). The mice were than shaved and secured in a stereotaxic instrument. The designated surgery area was swabbed and sterilized with 70% ethanol, and a sagittal section of the skin was made inferior to the Occiput. A capillary tube was inserted to the Cisterna Magna under the microscope, while the mouse head formed an obtuse (135o) angle with its body. The CSF was aspirated from the capillary to a pre-marked tube using a 3-ml syringe. The tube was immediately frozen in liquid nitrogen and was later stored in -80°C. The CSF samples were analyzed for glutamate and GABA concentrations. Glutamate and GABA were measured using high performance liquid chromatography with fluorescence detection (HPLC-FD, 250 × 4.6 mm, 2.5 µl particle size column, Hypersyl Gold 5U column, Thermo Scientific, USA). The excitation and emission wavelengths were 340 and 450 nm, respectively. The amino acid standards for glutamate and GABA (Sigma-Aldrich) or samples were precolumn derivatized with OPA reagent solution. The derivatization reagent was prepared as follows: 9mg o-phthalaldehyde was dissolved in 167μl methanol, then 1.5ml 0.4M sodium borate and 7μl 2-mercaptoethanol were added and mixed well. The solution was stored at 4°C for two days and protected from light. Gradient elution was used to separate the mixture of compounds: mobile phase A consisted of 0.1M sodium acetate buffer (pH 6.7) while mobile phase B consisted of 97.5% methanol and 2.5% tetrahydrofuran, as we used previously^1^. The flow rate of the pump was set at 1.4 ml/min. The amino acid concentration was determined using the peak area and the external standard method.

- 1. Evaluation of cannabinoids in serum

Whole blood samples were collected after sacrificing the mice by terminal bleeding.

The whole blood samples were incubated at room temperature for 30 minutes, and afterwards centrifuged at 1000 rcf for 10 minutes at 4°C. After centrifugation, the clear phase containing the serum was collected gently by pipetting and transferred to a new 1.5 ml tube for storage at -80°C. Experimental analysis of cannabinoids was carried out using LC-MS/MS system which consisted of binary UPLC (Nexera X2, Shimadzu) coupled to QTRAP 6500+ tandem mass spectrometer (SCIEX). The cannabinoids and isotopically labeled internal standards (2-arachidonoyl glycerol, 2-arachidonoyl glycerol-D5, arachidonoyl ethanolamide, arachidonoyl ethanolamide-D4) were purchased from Cayman Chemical. The LC-MS analysis method and sample preparation protocol were developed in Interdepartmental Analytical, The Robert H. Smith Faculty of Agriculture, Food and Environment, The Hebrew University of Jerusalem.

- 1. RNA sequencing of cerebellar brain samples

RNA from the right cerebellum of InsG3680 *Shank3* mutant mice was extracted using RNeasy Mini Kit (79254, Qiagen, Hilden, Germany). In short, mice designated for brain dissection were sacrificed using CO2 one hour post oral gavage. Brains were removed quickly and dissected on ice for right cerebellum: the pons was separated from the brain using forceps. Immediately afterwards the cerebellum was dissected from the cortex using a scalpel. Then, the cerebellum was dissected in the midline and the right half was snap frozen in liquid nitrogen, then transferred to -80°C until RNA extraction.

RNA extraction was performed following the RNeasy mini kit protocol. Afterwards, RNA sequencing was performed by Macrogen Europe NGS services. Raw sequencing data was trimmed and aligned to the GRCm38 assembly using STAR 2.7.3a^2^. Differential gene expression analysis was performed in R 3.6.3, using DESeq2 1.26^3^ and surrogate variable analysis with sva 3.34. Clustering of the samples was assessed using the principle component analysis (PCA) of variance stabilize transformed data^4^. A single outlier sample was omitted, resulting in decent clustering with notable batch effect (Figure S1A). The batch effect was empirically corrected using surrogate variables produced using the sva package for R (v3.38), then incorporated to DESeq as covariates (Figure S1B). The improved clustering is visualized using limma's removeBatchEffect (Figure S1C) (limma v3.46.0)^5^.Raw data tables are supplied in supplementary materials (Tables S2-S3). Principal component analysis (PCA) is presented in supplementary (Figure S1).

- 1. Statistical analysis

The results are expressed as means± standard error mean (SEM). Statistical analysis was performed using unpaired Student’s t test for the direct comparison between two groups. Statistical analysis of data sets was carried out with the aid of GraphPad Prism 6.01 for Windows (Graphpad Software, CA, USA). Outliers were defined using GraphPad Prism, exploiting the ESD method (extreme studentized deviate, alpha=0.05).

**Supplementary materials and methods – bibliography**

1. Liu, P. *et al.* Uridine decreases morphine-induced behavioral sensitization by decreasing dorsal striatal dopamine release possibly via agonistic effects at GABA A receptors. *Eur. Neuropsychopharmacol.* **24**, 1557–1566 (2014).

2. Dobin, A. *et al.* STAR: Ultrafast universal RNA-seq aligner. *Bioinformatics* **29**, 15–21 (2013).

3. Love, M. I., Huber, W. & Anders, S. Moderated estimation of fold change and dispersion for RNA-seq data with DESeq2. *Genome Biol.* **15**, 1–21 (2014).

4. Huber, W., Von Heydebreck, A., Sültmann, H., Poustka, A. & Vingron, M. Variance stabilization applied to microarray data calibration and to the quantification of differential expression. *Bioinformatics* **18**, (2002).

5. Ritchie, M. E. *et al.* Limma powers differential expression analyses for RNA-sequencing and microarray studies. *Nucleic Acids Res.* **43**, e47 (2015).
