# Supplementary material for: Behavioral aspects and neurobiological properties underlying medical cannabis treatment in Shank3 mouse model of autism spectrum disorder
Source: Transl Psychiatry. 2021 Oct 13;11:524. doi: 10.1038/s41398-021-01612-3 (PMC8514476; doi:10.1038/s41398-021-01612-3)
Supplement: Supplementary file 3 — Suppelmentary table 1- cannabis oils HPLC reports [file 41398_2021_1612_MOESM3_ESM.docx]

| **Name of product: Avidekel oil 5mg/ml** |  |
| --- | --- |
| **Batch Number:** |  |
| **Lab No.:** **TOL-19-040** |  |
| **Date of analysis: 24.1.19** |  |

| **Test** | **Specifications** | | **Results %** | **Passed/Failed** |
| --- | --- | --- | --- | --- |
| **HPLC Assay** | | | | |
| CBDA | For Information Only | | <0.1 |  |
| **CBD** | For Information Only | | 0.50 |  |
| **HPLC Related Substances** | | | | |
| THCA | | For Information Only | <0.1 |  |
| **THC^△9^** | | For Information Only | <0.1 |  |
| CBDV | | For Information Only | <0.1 |  |
| CBC | | For Information Only | <0.1 |  |
| CBG | | For Information Only | <0.1 |  |
| THC^△8^ | | For Information Only | <0.1 |  |
| **CBN** | | For Information Only | <0.1 |  |

tTHC = 0.877*[THCA] + [THC]

tCBD = 0.877*[CBDA] + [CBD]

Approved by: Tsiona Nehemia Signature: ________________ Date: 4.2.19

| **Name of product: ERZ oil 1mg/ml** |  |
| --- | --- |
| **Batch Number:** |  |
| **Lab No.: TOL-19-043** |  |
| **Date of analysis:**  **24.1.19** |  |

***Assay By HPLC***

| **Test** | **Specifications** | | **Results %** | | **Passed/Failed** |
| --- | --- | --- | --- | --- | --- |
| **HPLC Assay** | | | | | |
| THCA | For Information Only | | | <0.1 |  |
| **THC^△9^** | For Information Only | | | 0.1 |  |
| **HPLC Related Substances** | | | | | |
| CBDA | | For Information Only | | <0.1 |  |
| **CBD** | | For Information Only | | <0.1 |  |
| CBDV | | For Information Only | | <0.1 |  |
| CBC | | For Information Only | | <0.1 |  |
| CBG | | For Information Only | | <0.1 |  |
| THC^△8^ | | For Information Only | | <0.1 |  |
| **CBN** | | For Information Only | | <0.1 |  |

tTHC = 0.877*[THCA] + [THC]

tCBD = 0.877*[CBDA] + [CBD]

Approved by: Tsiona Nehemia Signature: ________________ Date: 4.2.19

| **Name of product: Pure CBD oil 5mg/ml** |  |
| --- | --- |
| **Batch Number:** |  |
| **Lab No.:** **TOL-19-045** |  |
| **Date of analysis: 24.1.19** |  |

| **Test** | **Specifications** | | **Results %** | **Passed/Failed** |
| --- | --- | --- | --- | --- |
| **HPLC Assay** | | | | |
| CBDA | For Information Only | | <0.1 |  |
| **CBD** | For Information Only | | 0.5 |  |
|  | | | | |
| THCA | | For Information Only | <0.1 |  |
| **THC^△9^** | | For Information Only | <0.1 |  |
| CBDV | | For Information Only | <0.1 |  |
| CBC | | For Information Only | <0.1 |  |
| CBG | | For Information Only | <0.1 |  |
| THC^△8^ | | For Information Only | <0.1 |  |
| **CBN** | | For Information Only | <0.1 |  |

tTHC = 0.877*[THCA] + [THC]

tCBD = 0.877*[CBDA] + [CBD]

Approved by: Tsiona Nehemia Signature: ________________ Date: 24.1.19
