# Supplementary material for: Behavioral aspects and neurobiological properties underlying medical cannabis treatment in Shank3 mouse model of autism spectrum disorder
Source: Transl Psychiatry. 2021 Oct 13;11:524. doi: 10.1038/s41398-021-01612-3 (PMC8514476; doi:10.1038/s41398-021-01612-3)
Supplement: Supplementary file 6 — Figure S0 - behavioral tests raw data [file 41398_2021_1612_MOESM6_ESM.pptx]

## Slide 1
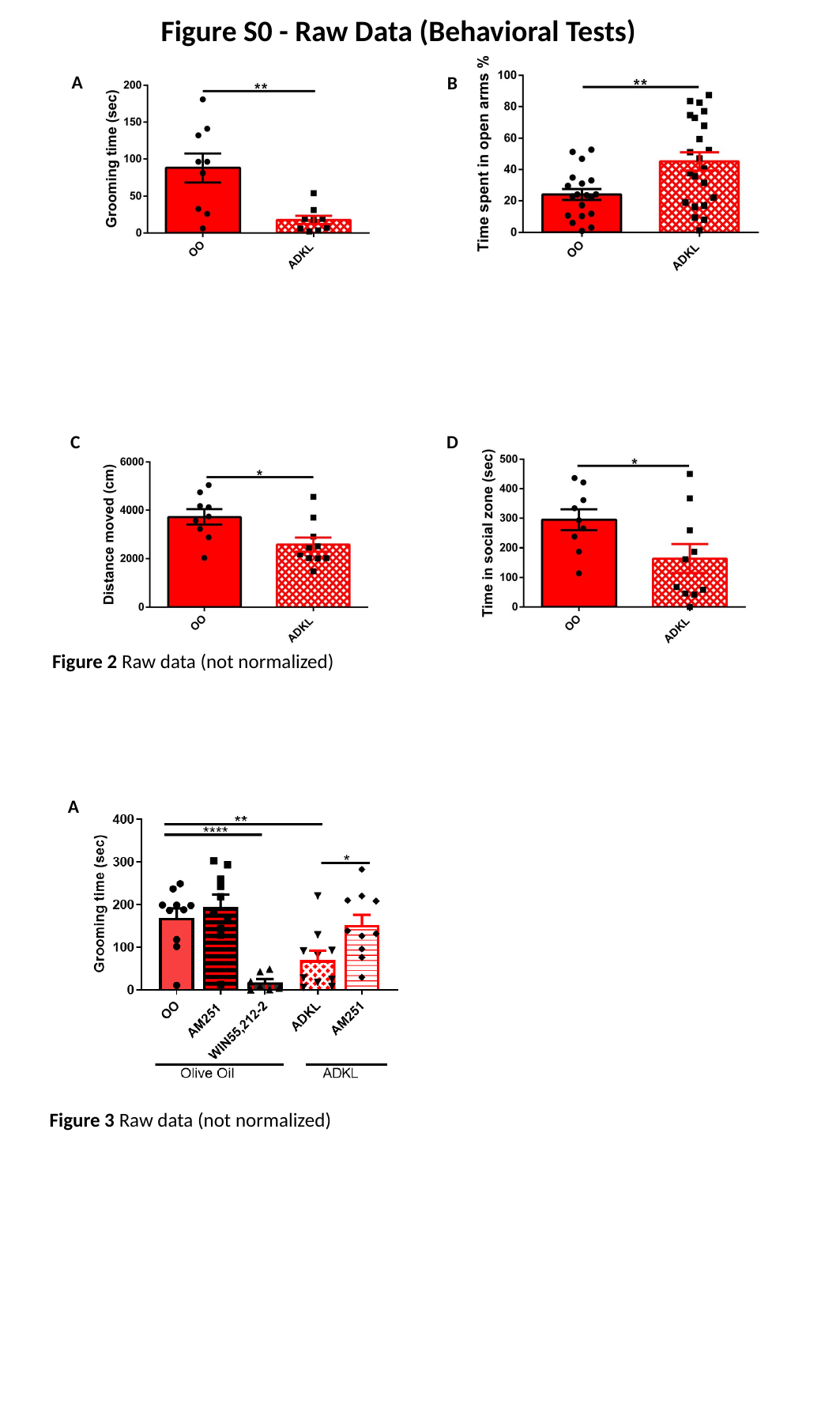

Figure S0 - Raw Data (Behavioral Tests)
A
B
C
D
Figure 2 Raw data (not normalized)
A
Figure 3 Raw data (not normalized)

## Slide 2
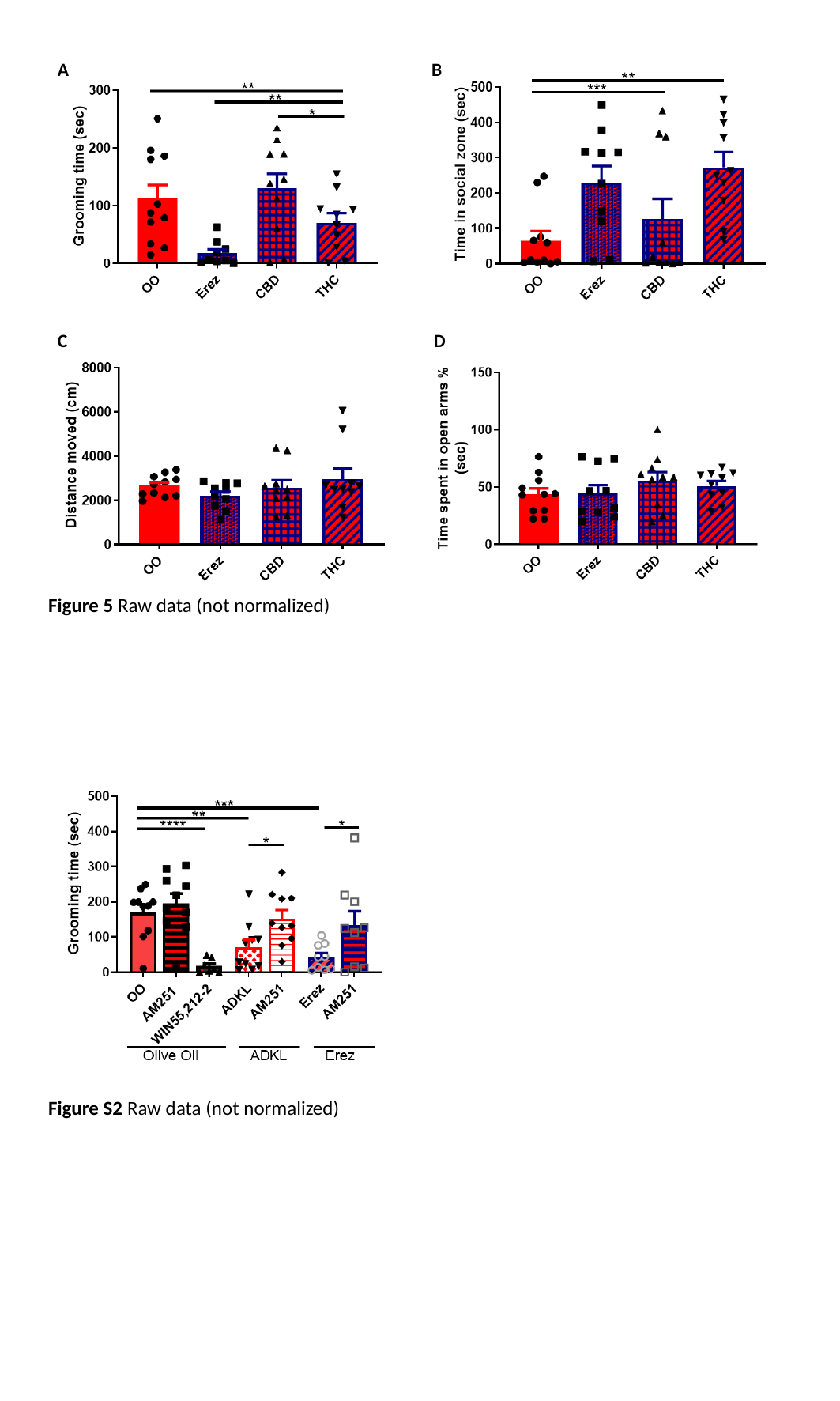

A
B
C
D
Figure 5 Raw data (not normalized)
Figure S2 Raw data (not normalized)

## Slide 3
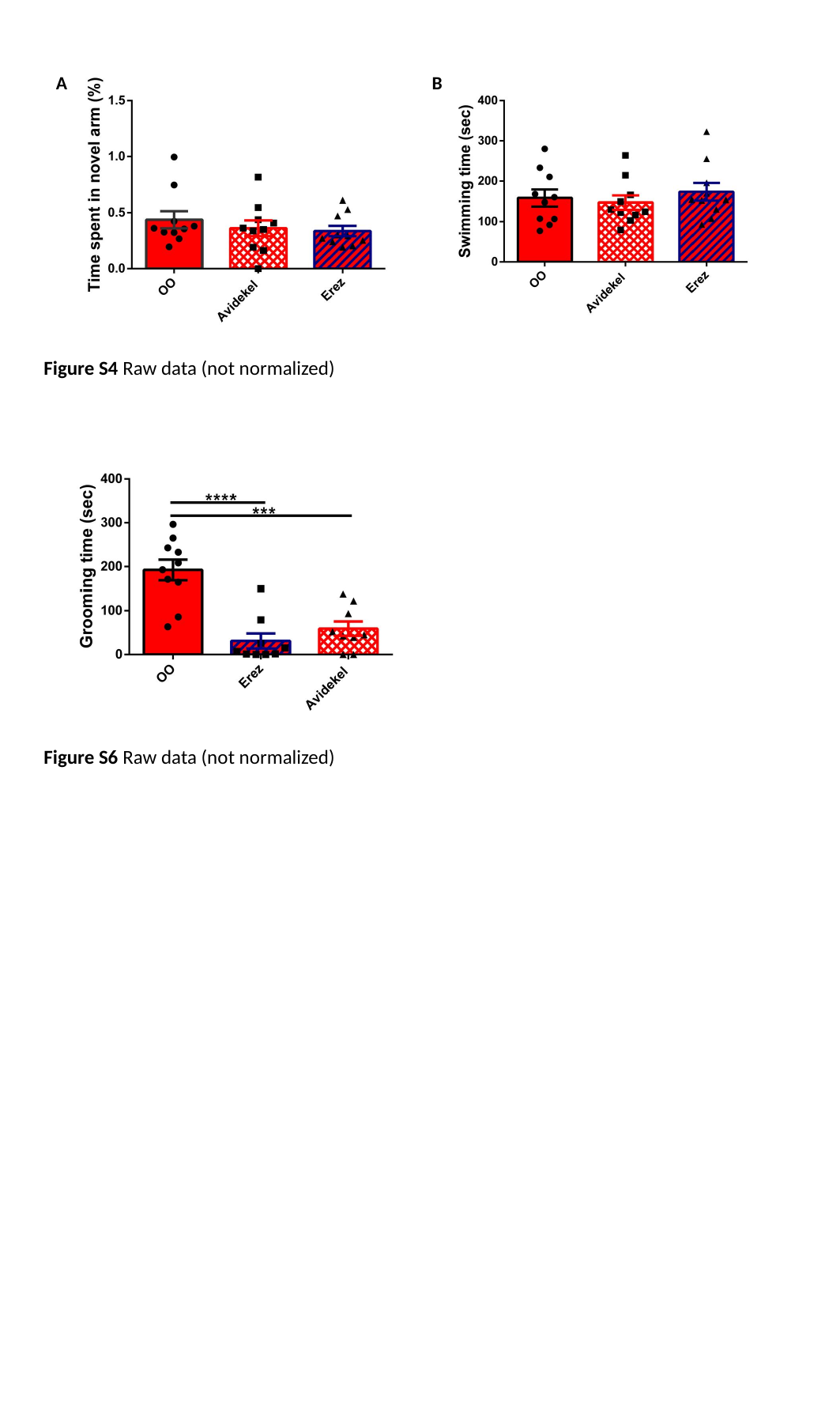

A
B
Figure S4 Raw data (not normalized)
Figure S6 Raw data (not normalized)
