# Supplementary figures and images for: Behavioral aspects and neurobiological properties underlying medical cannabis treatment in Shank3 mouse model of autism spectrum disorder
Source: Transl Psychiatry. 2021 Oct 13;11:524. doi: 10.1038/s41398-021-01612-3 (PMC8514476; doi:10.1038/s41398-021-01612-3)

## Slide 1
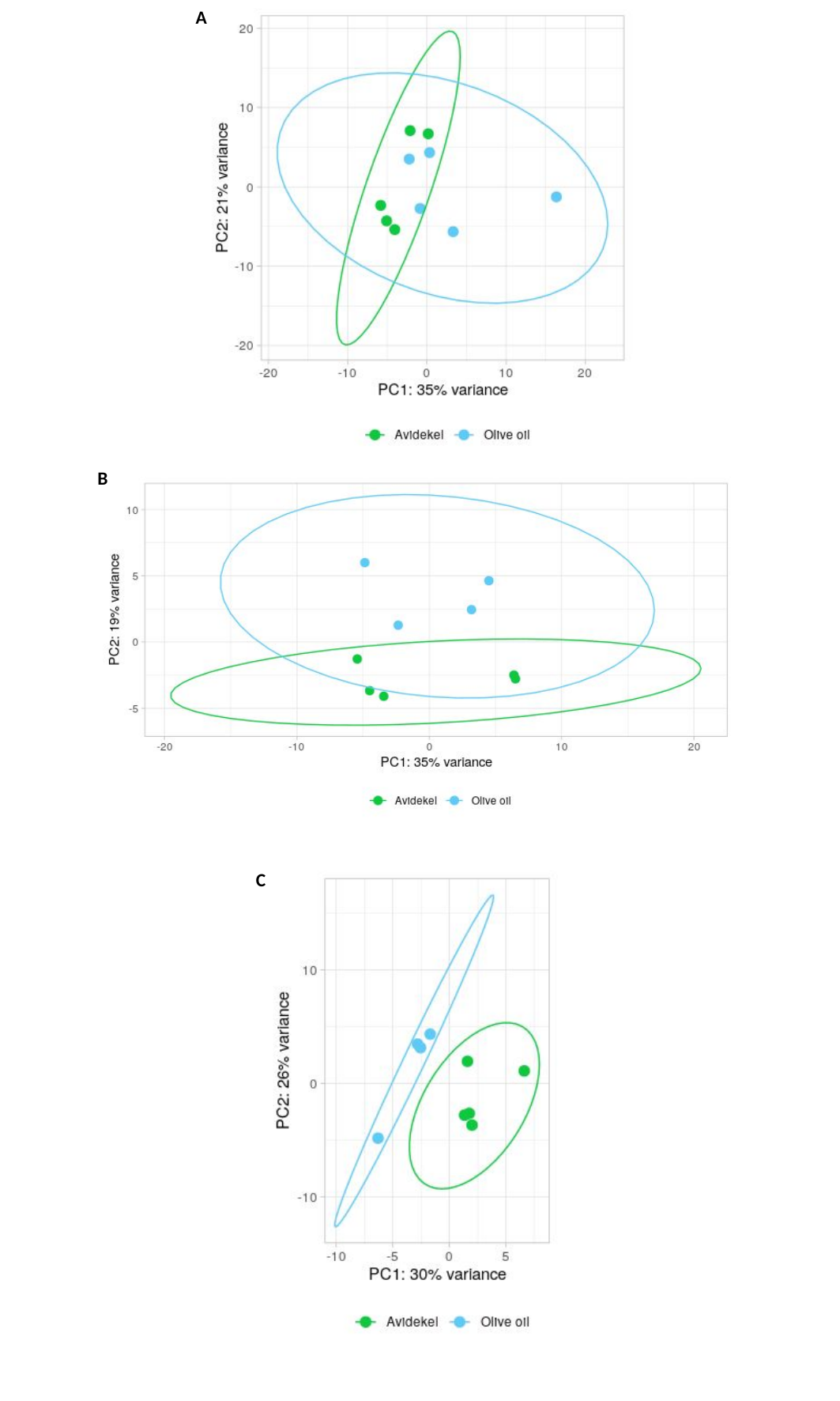

A
B
C

Supplement: Supplementary file 7 — Figure S1- PCA RNA-Seq [file 41398_2021_1612_MOESM7_ESM.pptx]

## Slide 1
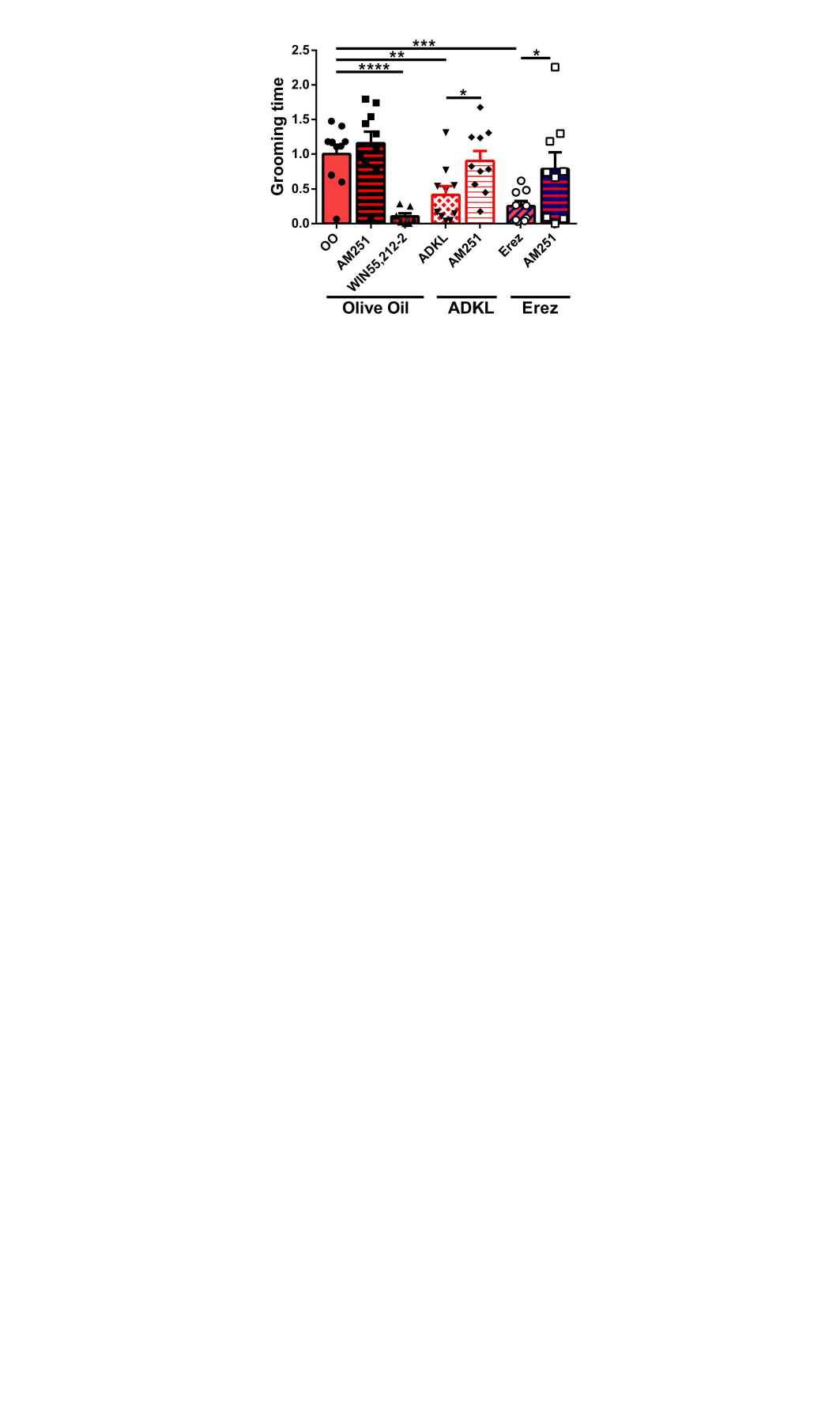

Supplement: Supplementary file 8 — Figure S2 - Erez CB1R blockade [file 41398_2021_1612_MOESM8_ESM.pptx]

## Slide 1
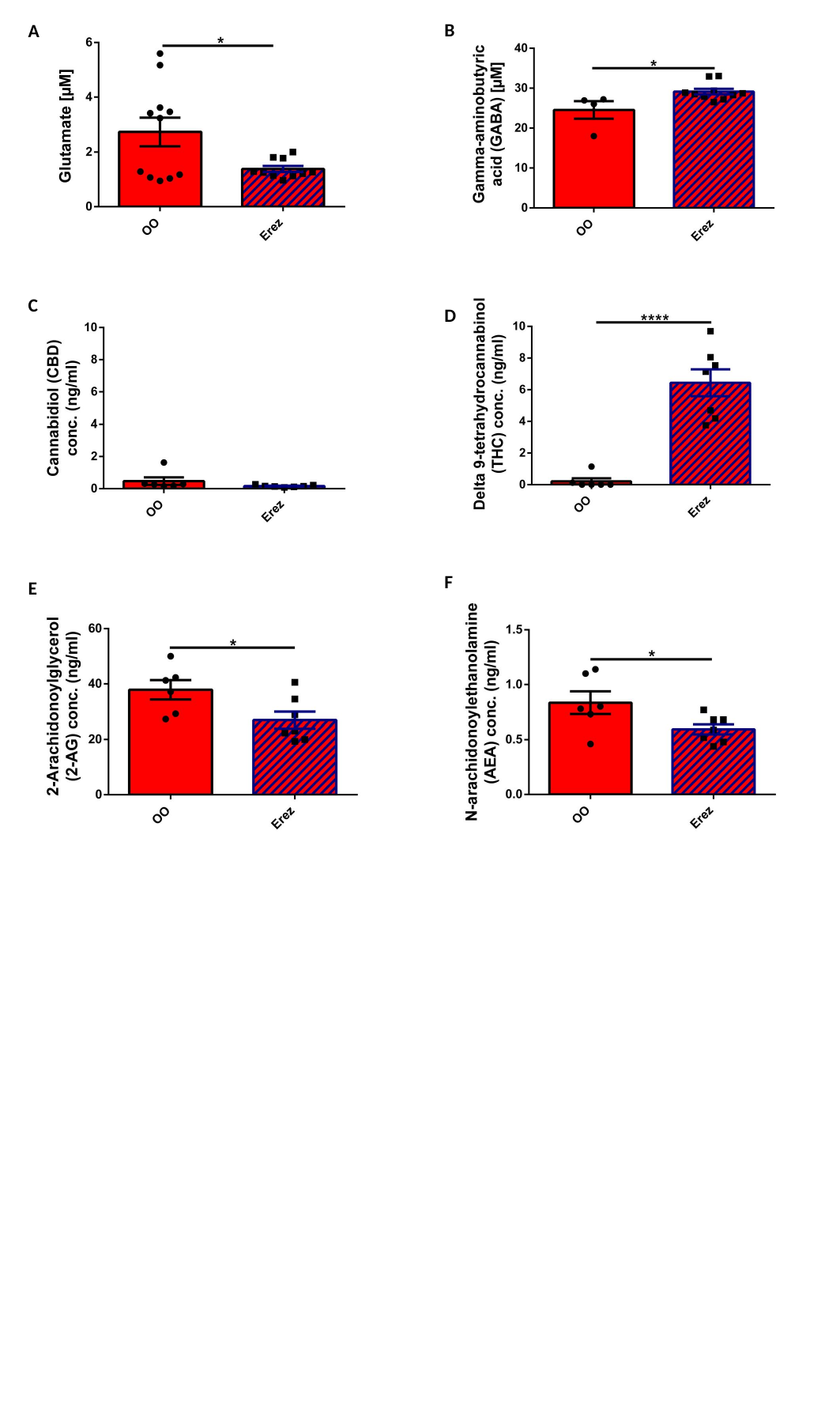

B
A
C
D
F
E

Supplement: Supplementary file 9 — Figure S3 - Erez Glu GABA CSF and serum cannabinoids [file 41398_2021_1612_MOESM9_ESM.pptx]

## Slide 1
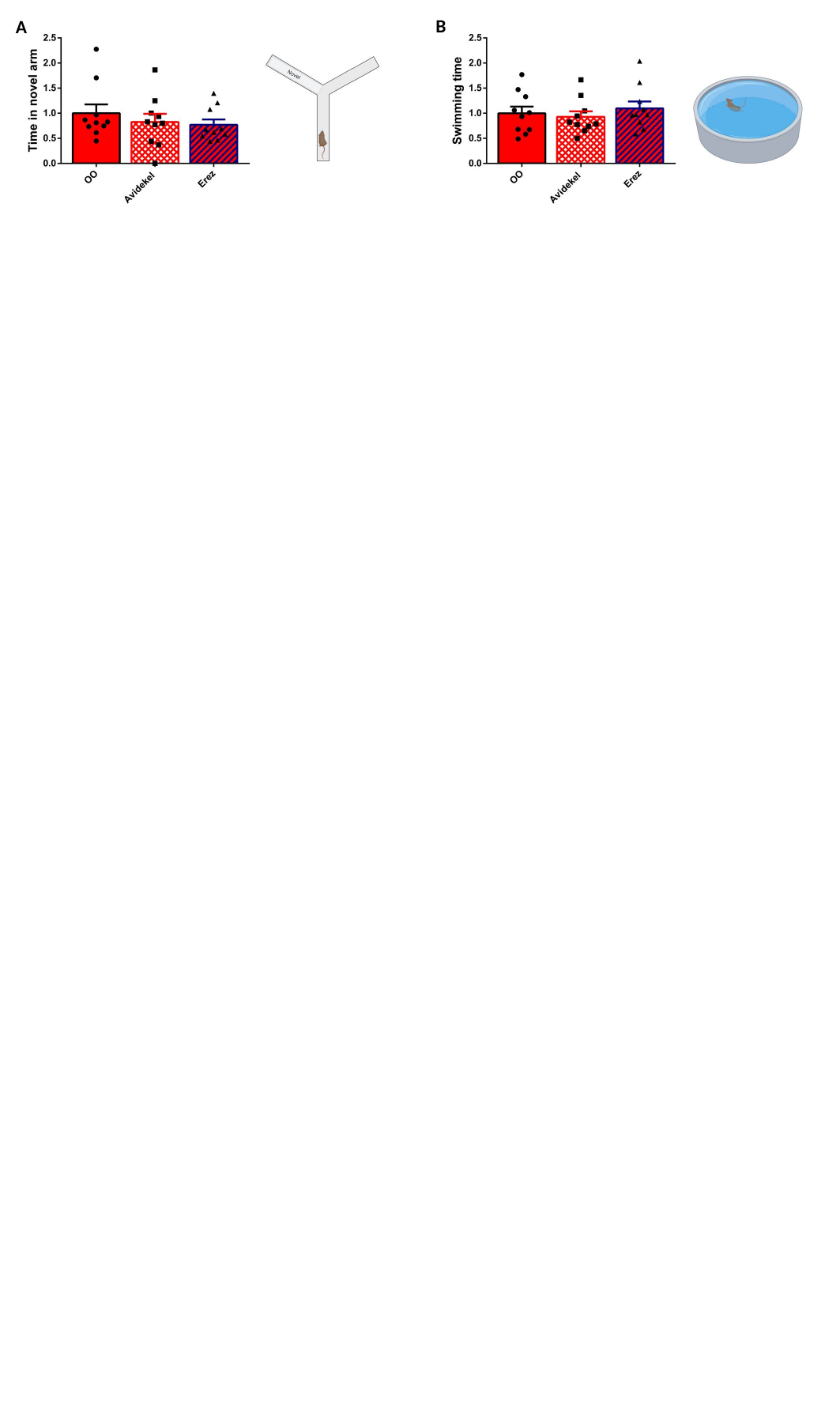

B
A

Supplement: Supplementary file 10 — Figure S4 - well being after treatment with medical cannabis [file 41398_2021_1612_MOESM10_ESM.pptx]

## Slide 1
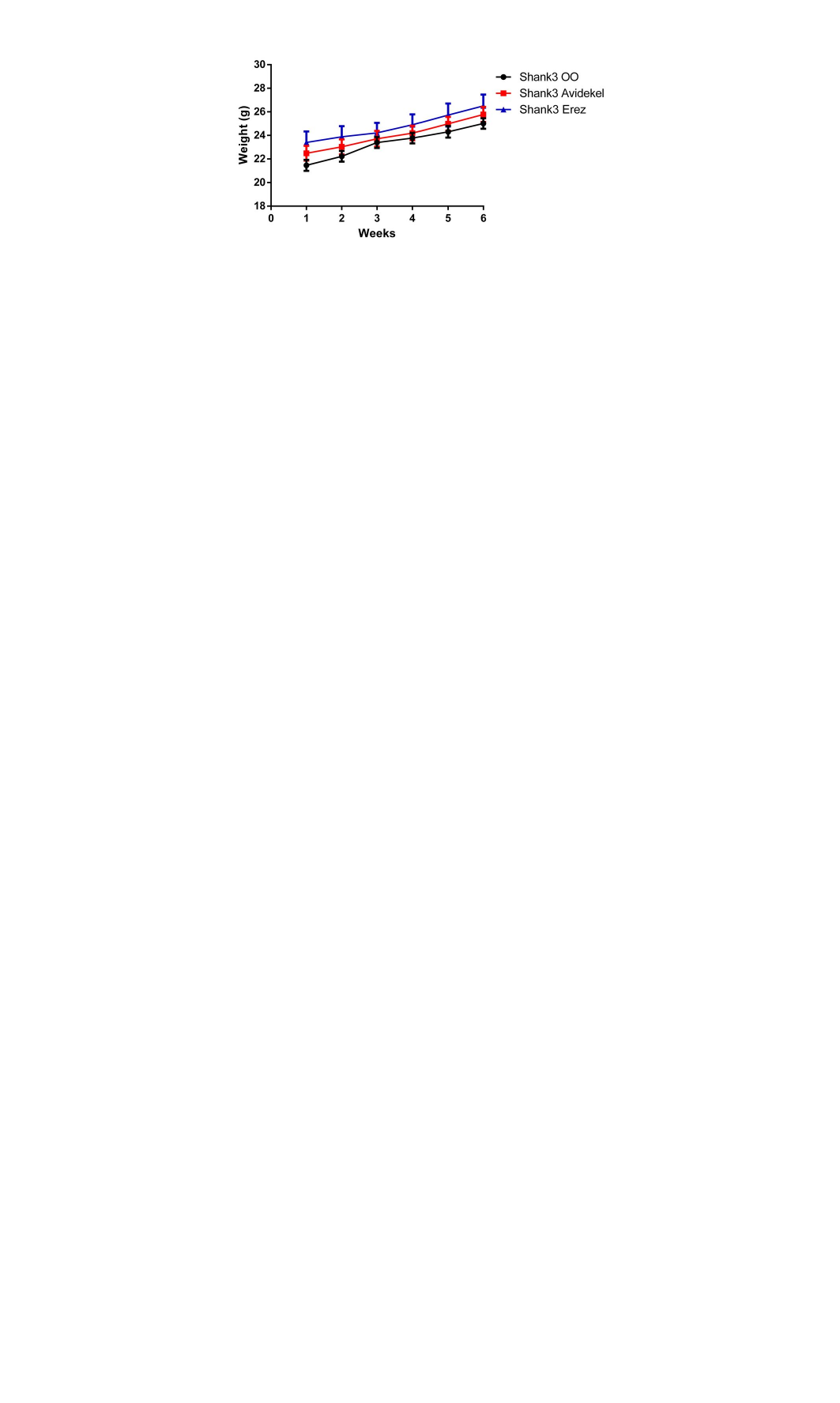

Supplement: Supplementary file 11 — Figure S5 - weight [file 41398_2021_1612_MOESM11_ESM.pptx]

## Slide 1
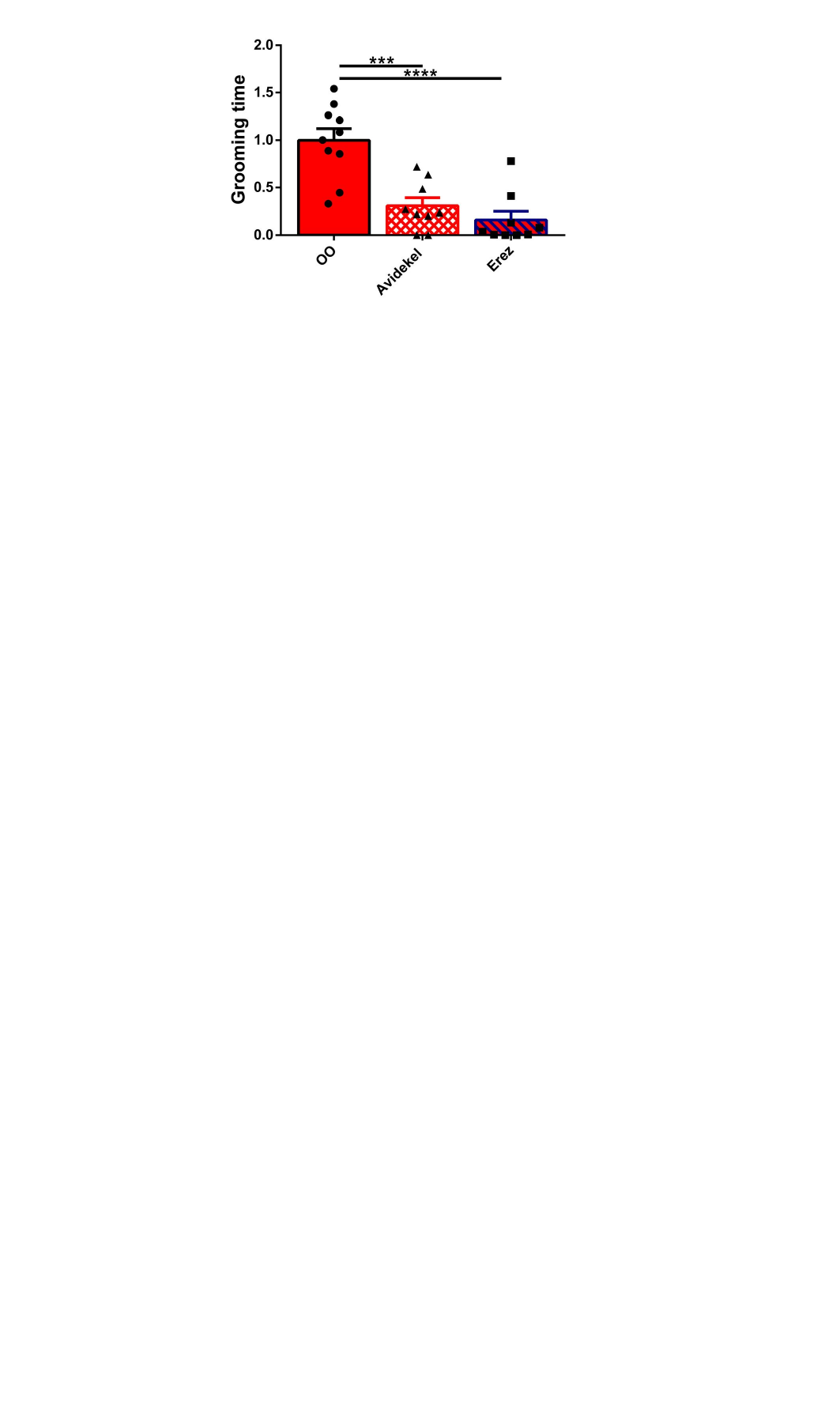

Supplement: Supplementary file 12 — Figure S6 - acute treatment only [file 41398_2021_1612_MOESM12_ESM.pptx]
