# Supplementary material for: Behavioral aspects and neurobiological properties underlying medical cannabis treatment in Shank3 mouse model of autism spectrum disorder
Source: Transl Psychiatry. 2021 Oct 13;11:524. doi: 10.1038/s41398-021-01612-3 (PMC8514476; doi:10.1038/s41398-021-01612-3)
Supplement: Supplementary file 13 — Supplementary legends [file 41398_2021_1612_MOESM13_ESM.docx]

**Figure S0 *Behavioral tests - raw data.***

**Figure S1 *CB1R mitigates the Erez-induced reduction in InsG3680 Shank3 mutant mice grooming behavior similarly to Avidekel oil.*** After treatment with CB1R antagonist AM-251 in addition to Avidekel or Erez oils, InsG3680 *Shank3* mutant mice have doubled the time they spent grooming, relative to their baseline grooming level, which was determined after receiving solely Avidekel or Erez oil. Furthermore, AM-251did not change the grooming behavior of the olive oil-treated InsG3680 *Shank3* mutant mice. In addition, treatment with the CB1R agonist Win 55,212-2 decreased significantly grooming behavior in olive oil-treated InsG3680 *Shank3* mutant mice. Results are normalized to the InsG3680 Shank3 olive oil control. Data are presented as mean ± SEM. N=29. *p<0.05, **p<0.01, ***p<0.001, ****p<0.0001. Two-tailed paired t-test between groups.

**Figure S2 *Erez oil treatment modifies the concentrations of neurotransmitters in the CSF as well as cannabinoids in the serum of InsG3680 Shank3 mutant mice.* (A-B)** HPLC-FD analysis of CSF neurotransmitters one hour post oral gavage. **(A)** Erez oil treatment caused a significant decrease of over 50% in glutamate concentration in the CSF of InsG3680 *Shank3* mutant mice **(B)**. Erez oil treatment led to increased GABA concentrations in the CSF of the treated mice. Data are presented as mean ± SEM. N=35 (glutamate: N=11 in olive oil group, N=10 in Erez group; GABA: N=4 in olive oil group, N=10 in Erez oil group). *p<0.05. Two-tailed t-test between groups. **(C-F)** LC-MS measurements of serum cannabinoids one hour post oral gavage treatment in InsG3680 *Shank3* mutant mice. Measurement of exocannabinoids CBD **(C)** and THC **(D)** concentrations in serum approved that Erez oil treatment contained undetectable amounts of CBD, with high concentrations of THC, as reported by the manufacturer. In addition, the concentrations of serum endocannabinoids 2-AG **(E)** and AEA **(F)** were significantly decreased after treatment with Erez oil, as compared to the olive oil control group. Data are presented as mean ± SEM. N=14 (7 in each group). *p<0.05, ****p<0.0001. Two-tailed t-test between groups.

**Figure S3 *Chronic treatment with medical cannabis oils did not impair the well-being of InsG3680 Shank3 mutant mice.* (A)** Treatment with Avidekel or Erez oil did not cause a significant decrease in the spatial working memory of InsG3680 *Shank3* mice in the Y-maze test. Results are normalized to the olive oil control group. Data are presented as mean ± SEM. N=20. Two-tailed t-test between groups. **(B)** Treatment with Avidekel or Erez oil did not cause a significant change in the susceptibility to negative mood of InsG3680 *Shank3* mutant mice, compared with the olive oil control group. Results are normalized to the olive oil control group. Data are presented as mean ± SEM. N=20. Two-tailed t-test between groups.

**Figure S4 *Chronic medical cannabis treatment did not affect the weight of InsG3680 Shank3 mutant mice.*** After six weeks of chronic oral gavage treatment, twice a week, with medical cannabis oils (Avidekel or Erez oils) there was no significant difference in the weight of treated InsG3680 *Shank3* mutant mice, compared to that of the olive oil treated control group. Data are presented as mean ± SEM. N=20. Two-tailed t-test between groups.

**Figure S5 *The beneficial effects of medical cannabis treatment on the repetitive grooming behavior of InsG3680 Shank3 mutant mice are acute.*** The acute effect of treatment with medical cannabis on the repetitive grooming behavior. InsG3680 *Shank3* mutant mice were treated once, one hour prior to the grooming behavioral test with either olive or medical cannabis oils. Treatment with medical cannabis oils decreased significantly the amount of repetitive grooming behavior performed by the treated InsG3680 *Shank3* mutant mice. Results are normalized to the olive oil group. Data are presented as mean ± SEM. N=20. ***p<0.0005, ****p<0.0001. Two-tailed t-test between groups.

**Figure S6** ***Principal component analysis (PCA) plot of variance stabilized counts.* (A)** Initially. **(B)** After removal of an outlier sample. **(C)** After batch correction (see methods section for full explanation).
